# Salmonella enterica Serovar Typhimurium Exploits Cycling through Epithelial Cells To Colonize Human and Murine Enteroids

**DOI:** 10.1128/mBio.02684-20

**Published:** 2021-01-12

**Authors:** Petra Geiser, Maria Letizia Di Martino, Pilar Samperio Ventayol, Jens Eriksson, Eduardo Sima, Anas Kh. Al-Saffar, David Ahl, Mia Phillipson, Dominic-Luc Webb, Magnus Sundbom, Per M. Hellström, Mikael E. Sellin

**Affiliations:** a Science for Life Laboratory, Department of Medical Biochemistry and Microbiology, Uppsala University, Uppsala, Sweden; b Department of Surgical Sciences, Uppsala University, Uppsala, Sweden; c Department of Medical Sciences, Gastroenterology and Hepatology Unit, Uppsala University, Uppsala, Sweden; d Department of Medical Cell Biology, Uppsala University, Uppsala, Sweden; University of Texas Southwestern Medical Center Dallas

**Keywords:** bioimaging, *Enterobacteriaceae*, enteroid, gastrointestinal infection, *Salmonella*

## Abstract

Enterobacterial pathogens infect the gut by a multistep process, resulting in colonization of both the lumen and the mucosal epithelium. Due to experimental constraints, it remains challenging to address how luminal and epithelium-lodged pathogen populations cross-feed each other *in vivo*. Enteroids are cultured three-dimensional miniature intestinal organs with a single layer of primary intestinal epithelial cells (IECs) surrounding a central lumen. They offer new opportunities to study enterobacterial infection under near-physiological conditions, at a temporal and spatial resolution not attainable in animal models, but remain poorly explored in this context. We employed microinjection, time-lapse microscopy, bacterial genetics, and barcoded consortium infections to describe the complete infection cycle of Salmonella enterica serovar Typhimurium in both human and murine enteroids. Flagellar motility and type III secretion system 1 (TTSS-1) promoted *Salmonella* Typhimurium targeting of the intraepithelial compartment and breaching of the epithelial barrier. Strikingly, however, TTSS-1 also potently boosted colonization of the enteroid lumen. By tracing the infection over time, we identified a cycle(s) of TTSS-1-driven IEC invasion, intraepithelial replication, and reemergence through infected IEC expulsion as a key mechanism for *Salmonella* Typhimurium luminal colonization. These findings suggest a positive feed-forward loop, through which IEC invasion by planktonic bacteria fuels further luminal population expansion, thereby ensuring efficient colonization of both the intraepithelial and luminal niches.

## Abstract (precis)

Pathogenic gut bacteria are common causes of intestinal disease. Enteroids—cultured three-dimensional replicas of the mammalian gut—offer an emerging model system to study disease mechanisms under conditions that recapitulate key features of the intestinal tract.

## INTRODUCTION

Salmonella enterica serovar Typhimurium is a common foodborne pathogen infecting the intestine of humans and other warm-blooded animals to cause acute enterocolitis. As a prototypic enteropathogen, *Salmonella* Typhimurium has been used to model the mechanisms underlying gut lumen colonization and the interplay with intestinal epithelial cells (IECs) and other mucosal cell types (1). Following ingestion of the pathogen, planktonic *Salmonella* Typhimurium expansion in the gut lumen and invasion of IECs both occur during the early phase of the infection (1). Flagellar motility and chemotaxis allow luminal *Salmonella* Typhimurium to penetrate the protective mucus layer and reach the epithelium, where the pathogen engages in near-surface swimming to scan for suitable target sites (2–6). Binding to the apical surface of IECs depends on bacterial adhesins and the syringe-like type III secretion system 1 (TTSS-1), encoded by *Salmonella* pathogenicity island 1 (SPI-1) (7–10). TTSS-1 subsequently induces bacterial uptake through transfer of a cocktail of effector proteins into the host cell (11–15). Hence, both flagella and TTSS-1 are critical virulence factors during gut colonization and drive IEC invasion both in tissue culture models and *in vivo* (1, 16).

Following internalization into IECs, *Salmonella* Typhimurium downregulates TTSS-1 and flagella and expresses a second TTSS (TTSS-2) encoded by SPI-2 to control intracellular trafficking and establish an intracellular niche (15, 17–19). The pathogen population expands within a vacuolar compartment referred to as the *Salmonella*-containing vacuole (SCV) (18, 20–23). Cytosolic hyperreplication, resulting from vacuolar escape, has also been reported in cultured epithelial cells (20, 21, 24). In addition, some IEC-invading *Salmonella* Typhimurium organisms can breach the epithelial barrier, thus initiating systemic bacterial spread (15, 17, 18, 25). However, the life span of the intraepithelial *Salmonella* Typhimurium population is limited by IEC-intrinsic, inflammasome-dependent detection and expulsion of infected IECs from the epithelium (17, 20, 22, 26, 27). Previous findings (17, 20) have hinted that bacterium-containing expelled IECs might contribute to *Salmonella* Typhimurium reseeding of the lumen, but the extent to which IEC invasion and luminal colonization are causally linked remains unclear.

Traditionally, *Salmonella* Typhimurium gut infection has been studied in *in vivo* models such as streptomycin-pretreated mice (28) or ligated bovine and rabbit ileal loops (29, 30) on one hand and in transformed/immortalized epithelial cell line cultures on the other (9, 12, 31). While they are physiologically relevant, the temporal resolution and control of experimental parameters remain poor in the *in vivo* models. In contrast, cell line infections allow stringent experimental control but lack three-dimensional (3D) tissue compartmentalization and primary cell behavior, and thus, they insufficiently model key aspects of the infection. A recent study revealed that the mechanistic basis for *Salmonella* Typhimurium invasion of IECs varies considerably between cell line infection models and the intact murine gut (11). This highlights the need to bridge the gap between physiological relevance and experimental simplicity when gut infectious diseases are being studied.

Gastrointestinal organotypic cultures containing primary epithelial cells provide a promising opportunity in this context. Such cultures can be established from pluripotent stem cells (referred to as PS-derived epithelial organoids) (32–35) or from adult stem cells residing in gastrointestinal crypts (resulting in exclusively epithelial structures termed gastroids, enteroids, or colonoids, depending on the segment of origin) (36–41). When grown in their 3D arrangement, these organotypic cultures feature a single-layered epithelium, encapsulating a central lumen that can be accessed by microinjection. Enterobacterial infections have recently been modeled in human PS-derived 3D organoids (42–45), murine 3D enteroids (46–48), and enteroid-derived 2D epithelial monolayers (49–54) or polarity-inverted 3D structures (55) that lack a luminal compartment. Experiments in human 3D gastroids have provided mechanistic insights into Helicobacter pylori infection (38, 56, 57). Microinjected human 3D enteroids, though recently used to investigate parasite infection (58, 59), have however remained virtually unexplored as a model for enterobacterial infection.

In this study, we validated microinjection of human and murine enteroids with fluorescent *Salmonella* Typhimurium as a 3D infection model with high temporal and spatial resolution. This permitted the tracing of both luminal and IEC-lodged *Salmonella* Typhimurium populations across the infection cycle by time-lapse microscopy. Using bacterial mutants, we identified flagellar motility as the main contributor to breaching of the epithelial barrier. In addition, our analyses established that cycles of TTSS-1-dependent IEC invasion, intraepithelial replication, and expulsion of infected IECs potently complement planktonic *Salmonella* Typhimurium growth for efficient colonization of the enteroid lumen.

## RESULTS

### Enteroid microinjection recapitulates key steps of the early *Salmonella* Typhimurium gut infection cycle.

**FIG 1:**
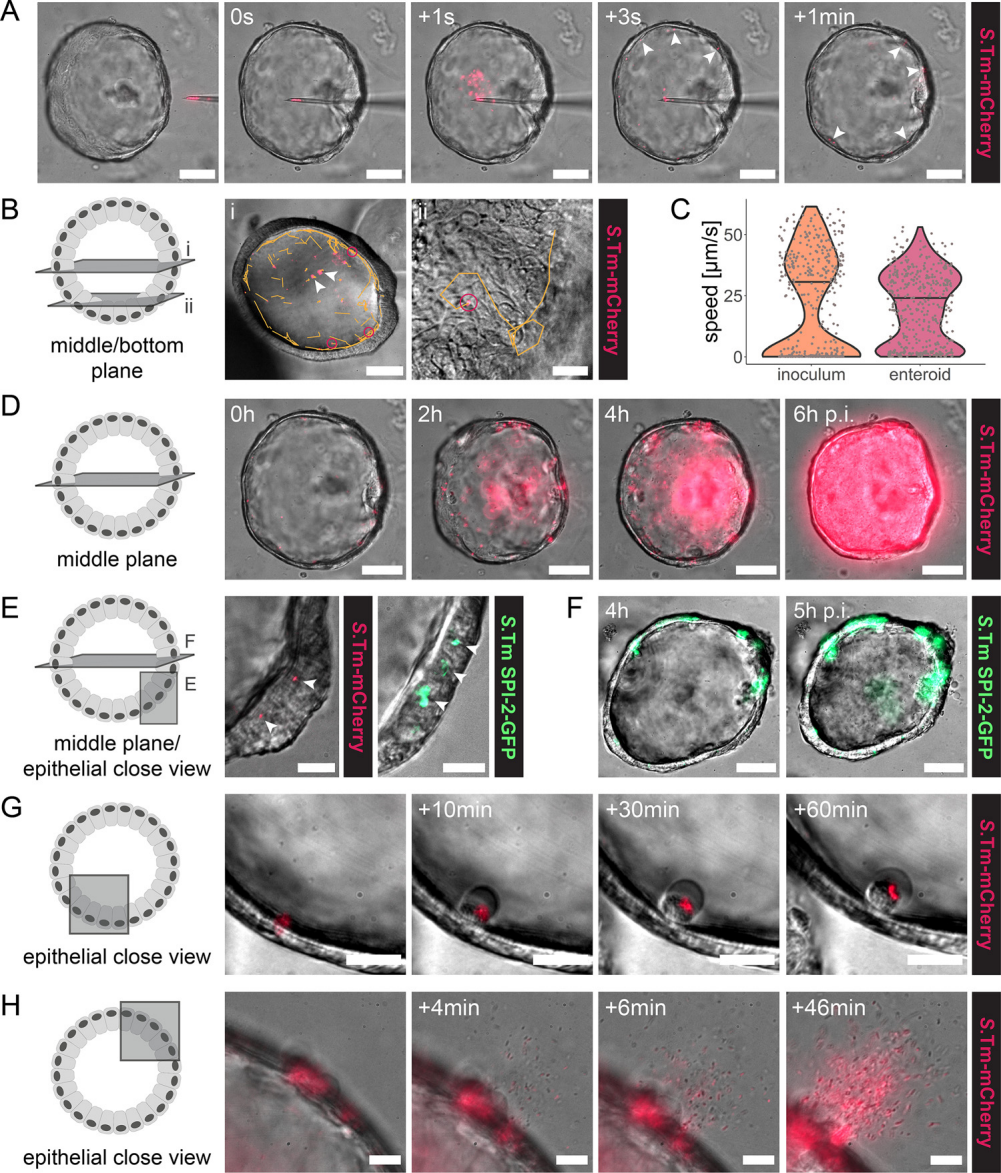
Recapitulation of the *Salmonella* Typhimurium early infection cycle in the human enteroid microinjection model. Human enteroids were injected with *Salmonella* Typhimurium (*S*.Tm) WT harboring the constitutive *rpsM*-mCherry or the SPI-2-inducible p*ssaG*-GFP reporter and imaged by wide-field differential interference contrast (DIC) and fluorescence time-lapse microscopy. (A) Microinjection procedure. Arrowheads indicate individual bacteria that have reached the epithelial surface. (B) Tracking of individual bacteria within the enteroid lumen. *Salmonella* Typhimurium organisms within the enteroid lumen, as well as a 1:700 to 1:1,000 dilution of the inoculum, were imaged at 300- to 500-ms intervals. The images show one frame of the time-lapse movie used for tracking. Orange lines indicate the complete tracks over the entire movie. Arrowheads mark nonmotile bacteria, whereas pink circles indicate examples of motile bacteria engaged in near-surface swimming. (C) Quantification of *Salmonella* Typhimurium motility in the inoculum and within the enteroid lumen based on bacterial tracking as shown in panel B. Each dot represents one bacterial track, and the horizontal line depicts the median. The data are based on >400 individual tracks per condition, originating from two independent experiments, in which motility was quantified in a total of nine enteroids. (D) Luminal expansion of the injected *Salmonella* Typhimurium population. (E) Examples of *Salmonella* Typhimurium invasion foci (arrowheads) based on constitutive (*rpsM*-mCherry; left) or SPI-2-inducible (p*ssaG*-GFP; right) reporters. (F) Intraepithelial expansion of SPI-2–GFP-positive invasion foci. (G) Expulsion of a *Salmonella* Typhimurium-containing IECs from the epithelial layer. (H) Breaching of the epithelial barrier and bacterial escape from the enteroid lumen, originating from an invasion focus. Bars, 50 μm (middle-plane view; A, B [i], D, and F) and 20 μm (bottom-plane and epithelial close view; B [ii], E, G, and H).

Early *Salmonella* Typhimurium infection involves luminal growth and IEC invasion. The steps of IEC invasion comprise (i) *Salmonella* Typhimurium flagellar motility, (ii) binding and TTSS-1-dependent invasion, (iii) intraepithelial replication within an SCV and/or in the cytosol, (iv) inflammasome-driven expulsion of infected IECs, and in some cases (v) bacterial breaching of the epithelial barrier. To validate enteroid microinjection as a model for these infection cycle events, human jejunum enteroids were established, injected with wild type (WT) *Salmonella* Typhimurium (SL1344) constitutively expressing mCherry (*rpsM-*mCherry) (60), and imaged by time-lapse microscopy (Fig. 1A; time-lapse movies related to the figures are available at https://doi.org/10.17044/scilifelab.12998570).

Microinjected *Salmonella* Typhimurium maintained flagellar motility, reached the epithelial surface within seconds after injection (Fig. 1A), and engaged in near-surface swimming (Fig. 1B). Tracking of individual bacteria revealed approximately similar levels of motile bacteria and average swimming speeds in the enteroid lumen and in the inoculum (Fig. 1B and C). Following injection, we observed a gradual increase in the numbers of fluorescent bacteria in the enteroid lumen, highlighting luminal replication over several hours post-injection (p.i.) (Fig. 1D). Luminal expansion with broadly similar kinetics was observed in spheroid human enteroids (Fig. 1D) and in morphologically more elaborate murine (C57BL/6 jejunum origin; two independently established enteroid lines) and human enteroids with clearly distinguishable crypt and villus domains (see Fig. S1 in the supplemental material). However, since microinjection of multilobulated enteroids was technically more challenging and quantification was more accurate in sphere-shaped enteroids with a clearly visible lumen, we used the latter for further analyses.

10.1128/mBio.02684-20.1 FIG S1 *Salmonella* Typhimurium colonization of multilobulated murine and human enteroids. Murine (C57BL/6, jejunum origin) and human enteroids were injected with *Salmonella* Typhimurium (*S*.Tm) WT *rpsM*-mCherry and imaged as for Fig. 1. (A to E) Murine enteroids were injected with various bacterial doses (mock-injected control; low dose, <20 *Salmonella* Typhimurium organisms per enteroid; intermediate dose, 20 to 50 organisms per enteroid; high dose, >50 organisms per enteroid), and enteroid colonization was quantified at 30-min intervals based on the increase in bacterial fluorescence within the enteroid relative to the initial intensity upon microinjection (0 h). (F and G) Murine enteroids independently established from a second mouse were injected with various *Salmonella* Typhimurium doses, and bacterial colonization was as quantified as in E. The examples shown in panel F are highlighted in pink in the quantification in panel G. (H and I) A multilobulated human enteroid was injected at intermediate dose, and colonization was quantified as for panel E. Each line in panels E, G, and I represents one enteroid. The grey lines in panel G depict enteroids not shown in the example images. The beginning of the dotted lines indicates the time point of bacterial escape from the enteroid lumen. The end of each line indicates the end of the respective time-lapse movie or the time point when the relative fluorescence intensity was decreasing again following bacterial escape from the enteroid lumen. Bars, 50 μm. MFI, mean fluorescence intensity. Download FIG S1, PDF file, 2.3 MB. Copyright © 2021 Geiser et al. 2021 Geiser et al. This content is distributed under the terms of the Creative Commons Attribution 4.0 International license.

*Salmonella* Typhimurium invasion foci within IECs were detected shortly after injection and continued to accumulate during the following hours (Fig. 1E, left). To investigate the timing of SCV establishment inside IECs, we injected enteroids with *Salmonella* Typhimurium expressing SPI-2-inducible green fluorescent protein (GFP) upon host cell invasion (p*ssaG*-GFP) (15). The appearance of GFP-positive intraepithelial foci at ∼3 to 4 h p.i. confirmed successful SCV establishment in some IECs (Fig. 1E, right; also, see Fig. S2A and B), and the expansion of these foci demonstrated intraepithelial replication (Fig. 1F). Applying a reporter induced upon access to the cytosolic metabolite glucose-6-phosphate (p*uhpT*-GFP) (18), we assessed the existence of *Salmonella* Typhimurium in the IEC cytosol. In agreement with recent observations in primary IECs by others (53), p*uhpT*-GFP-positive foci were few and barely detectable in the enteroid model (Fig. S2C). Furthermore, restriction of intraepithelial *Salmonella* Typhimurium included the frequent expulsion of bacterium-containing IECs into the enteroid lumen, detectable as early as ∼60 to 90 min p.i. (Fig. 1G; Fig. S2B and D). While the *Salmonella* Typhimurium population was contained within the epithelial lining during the first hours (Fig. 1D), breaching of the epithelial barrier was observed at later time points (Fig. 1H). Bacterial escape as a rule originated from an IEC invasion focus (Fig. 1H) and could be seen as a simple proxy for systemic *Salmonella* Typhimurium spread in the enteroid model.

10.1128/mBio.02684-20.2 FIG S2 *Salmonella* Typhimurium epithelial invasion and expulsion of infected IECs in microinjected enteroids. Human (A to D) and murine (E and F) enteroids were injected with *Salmonella* Typhimurium (*S*.Tm) WT p*ssaG*-GFP (A, B, E, and F) or p*uhpT*-GFP (C and D), respectively, and imaged as for Fig. 1. The intensity profiles in panels A, C, and E show the fluorescence intensity distribution along the lines indicated in the example images. Please note that the p*uhpT*-GFP signal is barely distinguishable from the surrounding background. Panels B, D, and F present examples of expulsion events involving IECs that harbor intracellular *Salmonella* Typhimurium based on the indicated fluorescent reporters. Bars, 20 μm. AU, arbitrary units. Download FIG S2, PDF file, 0.5 MB. Copyright © 2021 Geiser et al. 2021 Geiser et al. This content is distributed under the terms of the Creative Commons Attribution 4.0 International license.

Microbe-host interactions in organotypic models can exhibit considerable donor-to-donor variation (61–63). To exclude donor-specific effects, the *Salmonella* Typhimurium infectious cycle was replicated in a second independently established human enteroid line (Fig. S3). In addition, we repeated the experimental series in wild type murine enteroids. Also in this model, flagellar motility, near-surface swimming, luminal expansion, IEC binding and invasion, infected-IEC expulsion, and breaching of the epithelial barrier could be robustly detected and occurred with kinetics similar to in human enteroids (Fig. S2E and F; Fig. S4). Intraepithelial replication was observed to a lesser extent than in human enteroids, indicating that bacterial restriction within murine IECs might be even more efficient (Fig. S4E). Altogether, our findings confirm that human and murine enteroid microinjection recapitulate the predominant steps of the *Salmonella* Typhimurium gut infection cycle. The high temporal resolution enables dissection of how distinct events in the infection cycle are causally linked to each other.

10.1128/mBio.02684-20.3 FIG S3 The complete *Salmonella* Typhimurium infection cycle in an independently established human enteroid line. Human enteroids established independently from a second donor were injected with *Salmonella* Typhimurium (*S*.Tm) WT *rpsM*-mCherry (A to C, E, and F) or p*ssaG*-GFP (D) and imaged as for Fig. 1. (A) Mock-injected control showing that the microinjection procedure does not affect enteroid integrity or health during the experimental window used. (B) Luminal expansion of the injected *Salmonella* Typhimurium population. (C) *Salmonella* Typhimurium invasion foci within the epithelium of a microinjected enteroid. (D) Intraepithelial expansion of SPI-2–GFP-positive invasion foci. (E) Expulsion of a *Salmonella* Typhimurium-containing IEC from the epithelial layer. (F) Breaching of the epithelial barrier and bacterial escape from the enteroid lumen. Bars, 50 μm (A and B) and 20 μm (C and F). Download FIG S3, PDF file, 1.3 MB. Copyright © 2021 Geiser et al. 2021 Geiser et al. This content is distributed under the terms of the Creative Commons Attribution 4.0 International license.

10.1128/mBio.02684-20.4 FIG S4 Recapitulation of the *Salmonella* Typhimurium infection cycle in the murine enteroid microinjection model. Murine enteroids were injected with *Salmonella* Typhimurium (*S*.Tm) WT *rpsM*-mCherry (A to D, F, and G) or p*ssaG*-GFP (E) and imaged as for Fig. 1. (A) Tracking of individual bacteria in the inoculum and enteroid lumen as in Fig. 1B and C. The images show one frame of the time-lapse movie used for tracking. Orange lines indicate the complete tracks over the entire movie. Arrowheads mark nonmotile bacteria, whereas pink circles indicate examples of motile bacteria engaged in near-surface swimming. (B) Quantification of *Salmonella* Typhimurium motility in the inoculum and within the enteroid lumen based on bacterial tracking as shown in panel A. Each dot represents one bacterium, and the horizontal line depicts the median. The data are based on >400 individual tracks per condition originating from two independent experiments, in which motility was quantified in a total of 13 individual enteroids. (C) Luminal expansion of the injected *Salmonella* Typhimurium population. (D) Example of an individual *Salmonella* Typhimurium invading the epithelium of the injected enteroid. (E) Example of an SPI-2–GFP-positive invasion focus. The infected cell is expelled within minutes after appearance of the fluorescent signal. (F) Specific expulsion of *Salmonella* Typhimurium-containing IECs from the epithelial layer. Arrowheads in panels D, E, and F mark intraepithelial *Salmonella* Typhimurium invasion foci. (G) Breaching of the epithelial barrier and bacterial escape from the enteroid lumen originating from an invasion focus. Bars, 50 μm (middle-plane view; A [i] and C) and 20 μm (bottom-plane and epithelial close view; A [ii] and D to G). Download FIG S4, PDF file, 1.1 MB. Copyright © 2021 Geiser et al. 2021 Geiser et al. This content is distributed under the terms of the Creative Commons Attribution 4.0 International license.

### TTSS-1 boosts *Salmonella* Typhimurium colonization of IECs and the enteroid lumen.

We next addressed the contribution of *Salmonella* Typhimurium virulence factors to enteroid colonization. TTSS-1 encoded by *Salmonella* Typhimurium SPI-1 mediates translocation of effector proteins into host cells to promote bacterial invasion (16). To assess the impact of TTSS-1, human enteroids were injected with either fluorescently tagged *Salmonella* Typhimurium WT or a mutant deficient for a critical TTSS-1 structural component (Δ*invG*) (64) at different bacterial doses (low, <20 *Salmonella* Typhimurium organisms per enteroid; intermediate, 20 to 50 per enteroid; high, >50 per enteroid). Microinjected enteroids were followed by time-lapse microscopy, and colonization was quantified based on the increase in bacterial fluorescence within the enteroids over time.

**FIG 2:**
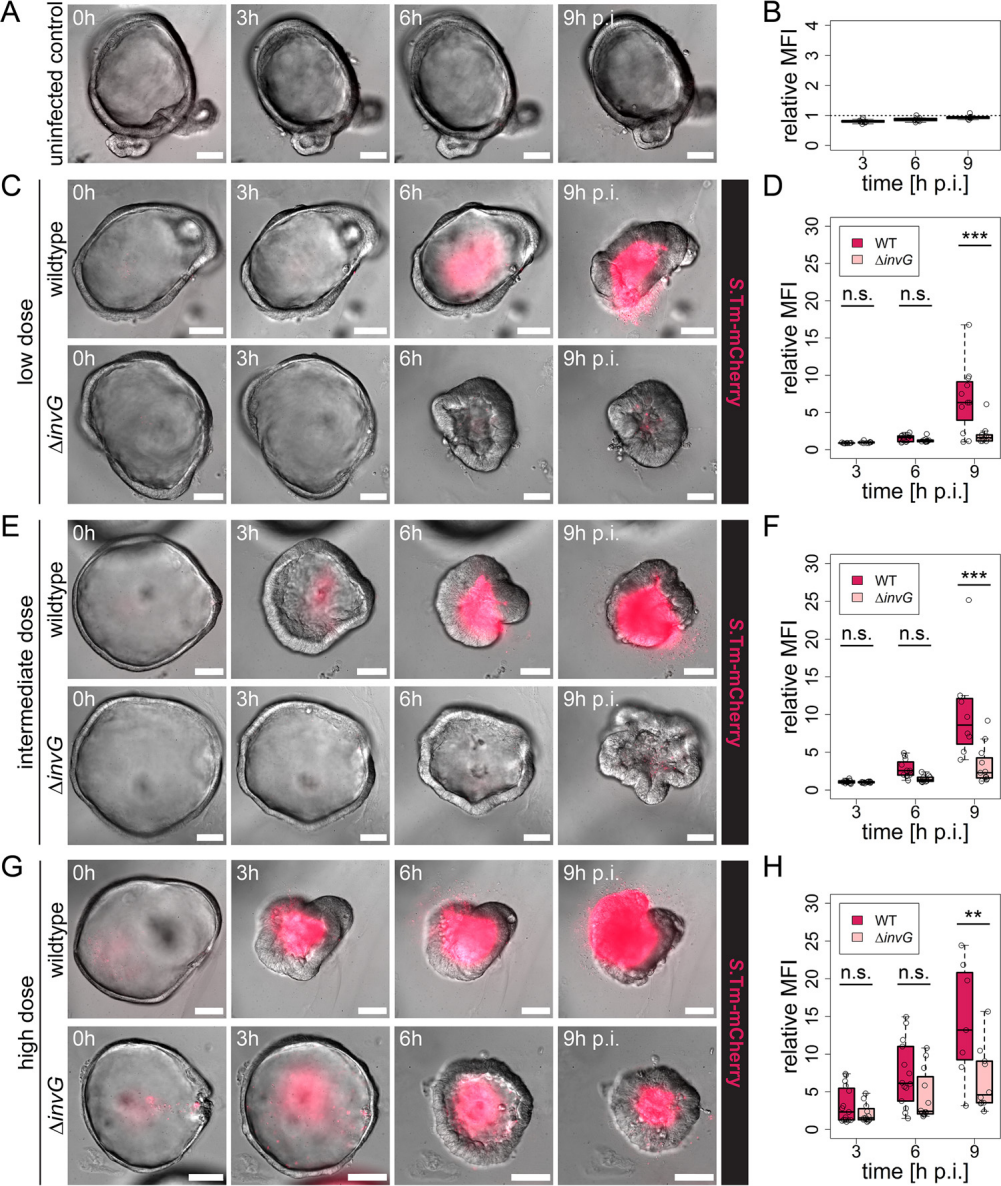
*Salmonella* Typhimurium TTSS-1 promotes human enteroid colonization. Human enteroids embedded within the same Matrigel dome were injected with *Salmonella* Typhimurium (*S*.Tm) WT or Δ*invG* carrying the constitutive *rpsM*-mCherry reporter at various bacterial doses: mock-injected control (A and B); low dose, <20 *Salmonella* organisms per enteroid (C and D); intermediate dose, 20 to 50 organisms per enteroid (E and F); and high dose, >50 organisms per enteroid (G and H). Enteroids were then imaged as for Fig. 1. Enteroid colonization was quantified at 3, 6, and 9 h p.i. based on the increase in bacterial fluorescence within the enteroid relative to the initial intensity upon microinjection (0 h). In the box plots in panels B, D, F, and H, the height of the boxes represents the interquartile range (IQR), whereas the horizontal line depicts the median. Whiskers extend to the most extreme data point but no further than 1.5× the IQR from the lower (first quartile) or upper (third quartile) boundary of the box. All data points are indicated as circles. The data for each dose are based on four independent experiments, in which enteroids embedded within the same Matrigel dome were injected with either strain. Data points for which the relative fluorescence intensity was decreasing again following bacterial escape from the enteroid lumen were excluded from analysis. Bars, 50 μm. Statistical significance was determined by two-way ANOVA with Tukey’s HSD *post hoc* test. n.s., nonsignificant; **, *P* < 0.01; ***, *P* < 0.001. MFI, mean fluorescence intensity.

Neither adverse effects of microinjection on enteroid health nor an increase in enteroid-associated fluorescence could be observed in mock-injected controls (Fig. 2A and B; Fig. S3A). This confirmed that an increase in fluorescence can be attributed to bacterial population expansion. Over time, injection of either *Salmonella* Typhimurium strain resulted in diffuse cytopathic effects, such as a variable degree of enteroid shrinkage, which was most consistently noted for *Salmonella* Typhimurium WT high-dose injections (Fig. 2C, E, and G). The kinetics of enteroid colonization for both *Salmonella* Typhimurium strains increased with the dose of bacteria injected (Fig. 2C to H; see Fig. S5A for individual curves). Notably, however, *Salmonella* Typhimurium WT expanded quickly and by 9 h p.i. had filled the enteroid lumen even upon low-dose injection. In contrast, the *Salmonella* Typhimurium Δ*invG* mutant population expanded significantly more slowly at all doses tested, and final fluorescence intensities reached only ∼1/3 to 1/2 of the level observed for *Salmonella* Typhimurium WT at 9 h p.i. (Fig. 2C to H). A similar attenuation of *Salmonella* Typhimurium Δ*invG* colonization capacity was observed also in murine enteroids (Fig. S5B and S6). These results were unexpected, since this TTSS-1-deficient strain has no detectable growth defect in rich medium (65) and since TTSS-1-deficient *Salmonella* Typhimurium can colonize the gut lumen of permissive mice (11, 15), although prior *in vivo* studies did not allow the high temporal resolution of the present experiments. As anticipated, we found that *Salmonella* Typhimurium Δ*invG* did not invade IECs upon enteroid microinjection (Fig. S7A). Moreover, a *Salmonella* Typhimurium strain maintaining an intact TTSS-1 but lacking four TTSS-1 effectors (SipA, SopB, SopE, and SopE2; referred to here as *Salmonella* Typhimurium Δ4) (66) both was incapable of invading IECs (Fig. S7B and C) and exhibited attenuated enteroid colonization kinetics similar to that of *Salmonella* Typhimurium Δ*invG* (Fig. S8). These observations led us to postulate that TTSS-1 activity boosts *Salmonella* Typhimurium colonization kinetics in enteroids by enabling IEC invasion.

10.1128/mBio.02684-20.5 FIG S5 Colonization of human and murine enteroids by *Salmonella* Typhimurium WT and *Salmonella* Typhimurium Δ*invG*. Human (A) or murine (B) enteroids were injected with *Salmonella* Typhimurium WT or *Salmonella* Typhimurium Δ*invG* carrying the constitutive *rpsM*-mCherry reporter at various bacterial doses, as specified in the legend to Fig. 2, and imaged as for Fig. 1. Enteroid colonization was quantified at 30-min intervals based on the increase in bacterial fluorescence within the enteroid relative to the initial intensity upon microinjection (0 h). The data are the same as in Fig. 2 (A; human enteroids) and Fig. S6 (B; murine enteroids) and are based on at least four independent experiments, in which enteroids embedded within the same Matrigel dome were injected with either strain. Each line represents one enteroid, and asterisks depict replicates where the relative fluorescence intensity exceeded 30. The beginning of the dotted lines indicates the time point of bacterial escape from the enteroid lumen. The end of each line indicates the end of the respective time-lapse movie or the time point when the relative fluorescence intensity was decreasing again following bacterial escape from the enteroid lumen. MFI, mean fluorescence intensity. Download FIG S5, PDF file, 1.2 MB. Copyright © 2021 Geiser et al. 2021 Geiser et al. This content is distributed under the terms of the Creative Commons Attribution 4.0 International license.

10.1128/mBio.02684-20.6 FIG S6 *Salmonella* Typhimurium TTSS-1 promotes murine enteroid colonization. Murine enteroids embedded within the same Matrigel dome were injected with *Salmonella* Typhimurium (*S*.Tm) WT or *Salmonella* Typhimurium Δ*invG* carrying the constitutive *rpsM*-mCherry reporter at various bacterial doses, as specified in the legend to Fig. 2, and imaged as for Fig. 1. Enteroid colonization was quantified at 2, 4, and 6 h p.i., as detailed for Fig. 2. In the box plots in panels B, D, F, and H, the height of the boxes represents the IQR, whereas the horizontal line depicts the median. Whiskers extend to the most extreme data point but no further than 1.5× the IQR from the lower (first quartile) or upper (third quartile) boundary of the box. All data points are indicated as circles. The data for each dose is based on at least four independent experiments, in which enteroids embedded within the same Matrigel dome were injected with either strain. Data points for which the relative fluorescence intensity was decreasing again following bacterial escape from the enteroid lumen were excluded from analysis. Asterisks in panels C and E indicate autofluorescence by epithelial cells. Bars, 20 μm. Statistical significance was determined by two-way ANOVA with Tukey’s HSD *post hoc* test. n.s., nonsignificant; *, *P* < 0.05; **, *P* < 0.01; ***, *P* < 0.001. MFI, mean fluorescence intensity. Download FIG S6, PDF file, 1.7 MB. Copyright © 2021 Geiser et al. 2021 Geiser et al. This content is distributed under the terms of the Creative Commons Attribution 4.0 International license.

10.1128/mBio.02684-20.7 FIG S7 *Salmonella* Typhimurium Δ*invG* and *Salmonella* Typhimurium Δ4 do not invade IECs upon enteroid microinjection. Human enteroids were injected with *Salmonella* Typhimurium (*S*.Tm) WT, *Salmonella* Typhimurium Δ*invG*, or *Salmonella* Typhimurium Δ4 carrying the constitutive *rpsM*-mCherry reporter and imaged as for Fig. 1. (A) Human enteroids embedded within the same dome were injected with either *Salmonella* Typhimurium WT or *Salmonella* Typhimurium Δ*invG* and followed by time-lapse microscopy for the appearance of invasion foci within the epithelium. (B and C) Human enteroids embedded within the same dome were injected with either *Salmonella* Typhimurium WT or *Salmonella* Typhimurium Δ4 and followed by time-lapse microscopy for the appearance of invasion foci within the epithelium. Accumulation of fluorescent *Salmonella* Typhimurium within the lumen at later time points was observed for all strains, whereas invasion foci were detected only for *Salmonella* Typhimurium WT. Arrowheads indicate intraepithelial invasion foci. Bars, 20 μm. Download FIG S7, PDF file, 0.3 MB. Copyright © 2021 Geiser et al. 2021 Geiser et al. This content is distributed under the terms of the Creative Commons Attribution 4.0 International license.

10.1128/mBio.02684-20.8 FIG S8 *Salmonella* Typhimurium TTSS-1 effectors promote human enteroid colonization. Human enteroids embedded within the same Matrigel dome were injected with *Salmonella* Typhimurium (*S*.Tm) WT or *Salmonella* Typhimurium Δ4 carrying the constitutive *rpsM*-mCherry reporter at various bacterial doses, as specified in the legend to Fig. 2, and imaged as for Fig. 1. Enteroid colonization was quantified at 3, 6, and 9 h p.i. as in Fig. 2. Due to the limited number of data points available for *Salmonella* Typhimurium WT at 9 h p.i., this time point had to be excluded from analysis at intermediate and high doses (F and H) (NA, not available). In the box plots in panels B, D, F, and H, the height of the boxes represents the IQR, whereas the horizontal line depicts the median. Whiskers extend to the most extreme data point but no further than 1.5× the IQR from the lower (first quartile) or upper (third quartile) boundary of the box. All data points are indicated as circles. The data for each dose are based on three independent experiments, in which enteroids embedded within the same Matrigel dome were injected with either strain. Data points for which the relative fluorescence intensity was decreasing again following bacterial escape from the enteroid lumen were excluded from analysis. Bars, 50 μm. Statistical significance was determined by two-way ANOVA with Tukey’s HSD *post hoc* test. n.s., nonsignificant; *, *P* < 0.05. MFI, mean fluorescence intensity. Download FIG S8, PDF file, 2.9 MB. Copyright © 2021 Geiser et al. 2021 Geiser et al. This content is distributed under the terms of the Creative Commons Attribution 4.0 International license.

### *Salmonella* Typhimurium flagellar motility is dispensable for enteroid colonization.

Flagellar motility is another key virulence determinant during early *Salmonella* Typhimurium infection of the intestine, allowing the bacterium to penetrate the mucus layer, reach the epithelial surface, and engage in TTSS-1-dependent IEC invasion *in vivo* (3, 4, 6). To assess the involvement of flagellar motility in enteroid colonization, we injected human and murine enteroids with fluorescent *Salmonella* Typhimurium WT or a nonmotile mutant lacking the flagellar motor protein MotA (Δ*motA*) but maintaining structurally intact flagella and quantified bacterial colonization kinetics.

**FIG 3:**
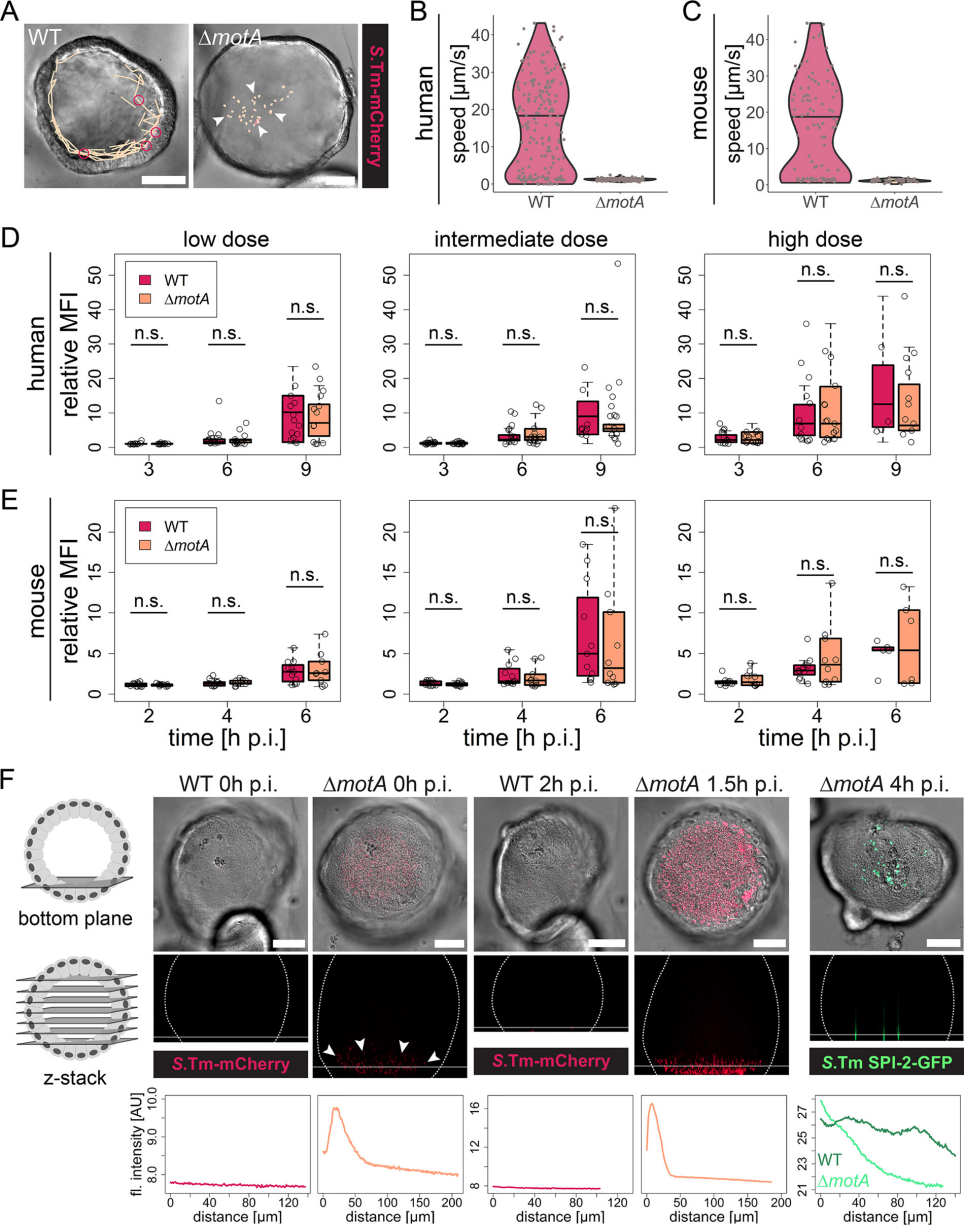
*Salmonella* Typhimurium flagellar motility is dispensable for human and murine enteroid colonization. Human and murine enteroids were injected with *Salmonella* Typhimurium (*S*.Tm) WT or Δ*motA* carrying the constitutive *rpsM*-mCherry reporter (A to F) or the SPI-2-inducible p*ssaG*-GFP reporter (F). (A) Tracking of individual *Salmonella* Typhimurium WT (left) or *Salmonella* Typhimurium Δ*motA* (right) within the human enteroid lumen, as in Fig. 1B. The images show one frame of the time-lapse movie used for tracking. Orange lines indicate the complete tracks over the entire movie. Arrowheads mark nonmotile bacteria, whereas pink circles indicate examples of motile bacteria engaged in near-surface swimming. Bars, 50 μm. (B and C) Quantification of *Salmonella* Typhimurium motility within the human (B) and murine (C) enteroid lumen based on bacterial tracking as shown in panel A. Each dot represents one bacterial track, and the horizontal line depicts the median. The data are based on >120 individual tracks per condition originating from two independent experiments, in which motility was quantified in a total of 13 enteroids injected with *Salmonella* Typhimurium WT and 7 enteroids injected with *Salmonella* Typhimurium Δ*motA* (B; human enteroids), or >50 individual tracks per condition originating from two independent experiments, in which a total of 10 enteroids injected with *Salmonella* Typhimurium WT and 3 enteroids injected with *Salmonella* Typhimurium Δ*motA* were analyzed. (D and E) Quantification of enteroid colonization 3, 6, and 9 h p.i. (D; human enteroids) or 2, 4, and 6 h p.i. (E; murine enteroids), as detailed in the legend to Fig. 2. The height of the boxes represents the IQR, whereas the horizontal line depicts the median. Whiskers extend to the most extreme data point but no further than 1.5× the IQR from the lower (first quartile) or upper (third quartile) boundary of the box. All data points are indicated as circles. The data for each species are based on five independent experiments, in which enteroids embedded within the same Matrigel dome were injected with either strain. Data points for which the relative fluorescence intensity was decreasing again following bacterial escape from the enteroid lumen were excluded from analysis. Statistical significance was determined by two-way ANOVA with Tukey’s HSD *post hoc* test. (F) Human enteroids embedded within the same dome were injected with *Salmonella* Typhimurium WT or *Salmonella* Typhimurium Δ*motA* carrying the constitutive *rpsM*-mCherry or the SPI-2-inducible p*ssaG*-GFP reporter, and confocal (*rpsM*-mCherry) or wide-field (p*ssaG*-GFP) z-stacks were acquired at the indicated time points p.i. Background subtraction was performed in Fiji for better visualization of the bacterial fluorescence. For the bottom plane, both the DIC and fluorescence channels are shown, whereas only the fluorescence channel is shown for the xz projection (z-stack). The dotted lines in the xz projections show the enteroid outline, and the horizontal line indicates the position of the bottom-plane image. The fluorescence intensity profiles along the *z* axis starting at the bottom of the enteroid (0 μm) are depicted below the respective images. In the rightmost graph, the fluorescence profile for an enteroid injected with the *Salmonella* Typhimurium WT is included for comparison. Bars, 50 μm. n.s., nonsignificant; MFI, mean fluorescence intensity; AU, arbitrary units.

The lack of motility was verified by tracking *Salmonella* Typhimurium WT and *Salmonella* Typhimurium Δ*motA* bacteria within the enteroid lumen. The quantification of *Salmonella* Typhimurium WT motility recapitulated our previous findings (Fig. 3A to C, WT; compare to Fig. 1C and Fig. S4B, enteroid), whereas no bacteria moving at a speed greater than 3 μm/s were detected for *Salmonella* Typhimurium Δ*motA* (Fig. 3A to C). Strikingly, however, no difference in enteroid colonization kinetics (based on the quantification of bacterial fluorescence intensities) was observed between the two strains at any bacterial dose or time point in human or murine enteroids (Fig. 3D and E; also, see Fig. S9 for individual curves). These findings surprisingly suggest that flagellar motility is not required for efficient bacterial colonization of enteroids.

10.1128/mBio.02684-20.9 FIG S9 Colonization of human and murine enteroids by *Salmonella* Typhimurium WT and *Salmonella* Typhimurium Δ*motA*. Human (A) or murine (B) enteroids were injected with *Salmonella* Typhimurium WT or *Salmonella* Typhimurium Δ*motA* carrying the constitutive *rpsM*-mCherry reporter at various bacterial doses, as specified in the legend to Fig. 2, and imaged as for Fig. 1. Enteroid colonization was quantified as for Fig. S5. The data are the same as in Fig. 3D and E and are based on five independent experiments, in which enteroids embedded within the same Matrigel dome were injected with either strain. Each line represents one enteroid. The beginning of the dotted lines indicates the time point of bacterial escape from the enteroid lumen. The end of each line indicates the end of the respective time-lapse movie or the time point when the relative fluorescence intensity was decreasing again following bacterial escape from the enteroid lumen. MFI, mean fluorescence intensity. Download FIG S9, PDF file, 1.2 MB. Copyright © 2021 Geiser et al. 2021 Geiser et al. This content is distributed under the terms of the Creative Commons Attribution 4.0 International license.

As we had found TTSS-1 to both drive IEC invasion and boost enteroid colonization, hence linking these two phenomena, we speculated that *Salmonella* Typhimurium Δ*motA* was able to successfully invade IECs after reaching the epithelial surface independent of flagellar motility. Therefore, we analyzed the vertical distribution of the bacterial population within the enteroid lumen by confocal microscopy. Flagellar motility of the *Salmonella* Typhimurium WT enabled rapid access to the epithelium (Fig. 1A to C), and consequently, no enrichment of bacteria at any specific location could be observed (Fig. 3F). In sharp contrast, *Salmonella* Typhimurium Δ*motA* displayed a clear accumulation atop the epithelium at the bottom plane of the enteroid, within minutes after inoculation (Fig. 3F). This local enrichment was further enhanced during the following ∼2 h p.i. (Fig. 3F). Finally, analysis of the vertical distribution of SPI-2-positive invasion foci following microinjection with *Salmonella* Typhimurium Δ*motA* p*ssaG*-GFP confirmed IEC invasion at the bottom plane at 4 h p.i. (Fig. 3F). This suggests that nonmotile *Salmonella* Typhimurium can reach the epithelial surface swiftly by gravitational sedimentation within enteroids, thereby allowing IEC invasion in the absence of functional flagella.

### Both TTSS-1 activity and flagellar motility promote *Salmonella* Typhimurium escape from enteroids.

**FIG 4:**
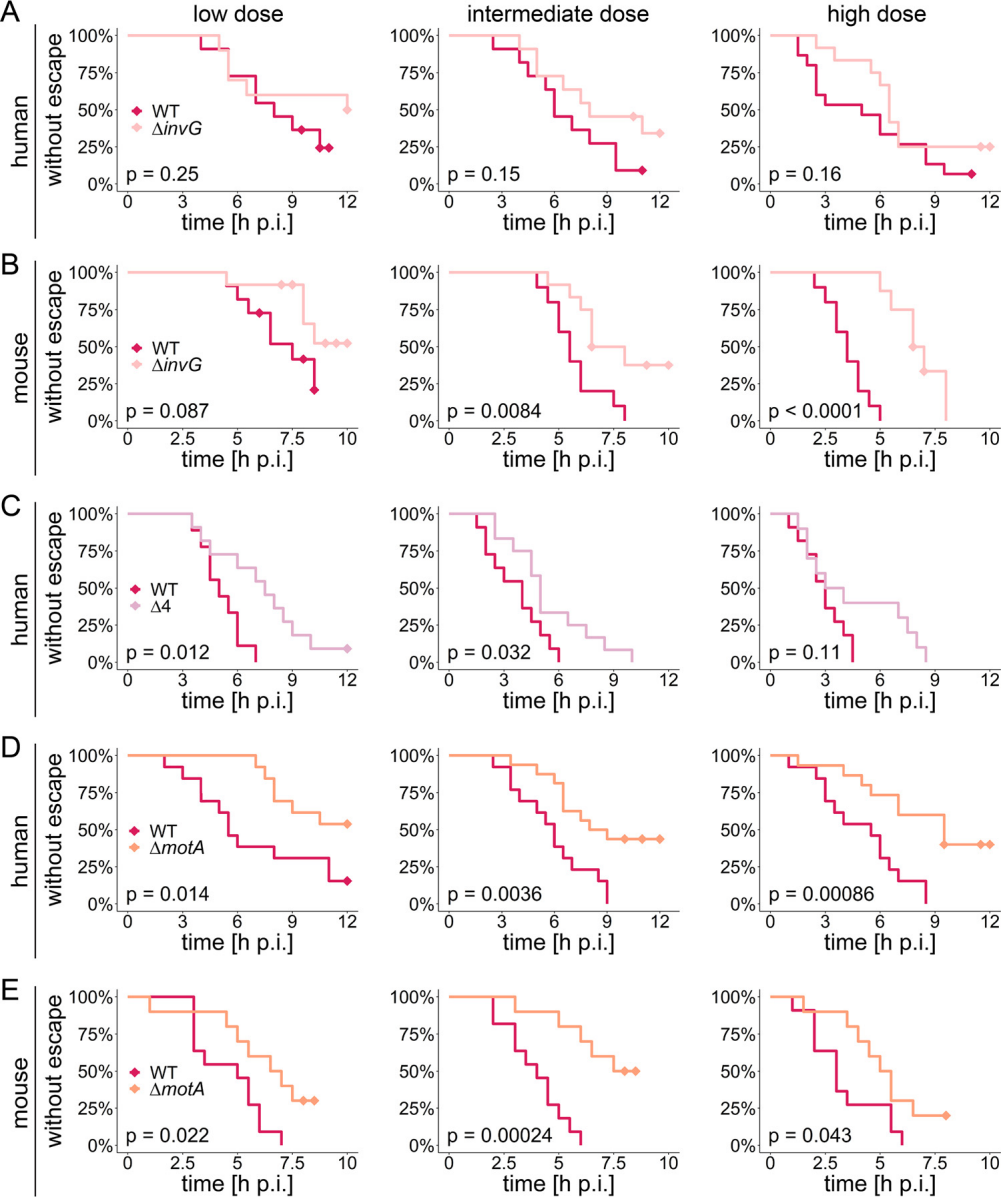
*Salmonella* Typhimurium TTSS-1 and flagellar motility promote breaching of the epithelial barrier in enteroids. Human (A, C, and D) and murine (B and E) enteroids were injected with *Salmonella* Typhimurium Δ*invG* (A and B), *Salmonella* Typhimurium Δ4 (C), or *Salmonella* Typhimurium Δ*motA* (D and E) carrying the constitutive *rpsM*-mCherry reporter at various bacterial doses (specified in the legend to Fig. 2) and imaged as for Fig. 1. Parallel injections with *Salmonella* Typhimurium WT *rpsM*-mCherry into enteroids within the same dome were performed for each strain and host species. The time until bacterial escape was quantified based on the Kaplan-Meier model. Diamonds indicate censored observations, i.e., enteroids in which *Salmonella* Typhimurium remained confined within the enteroid lumen until the end of the time-lapse movie. Please note that in some cases the movies ended prior to the experimental endpoint of ∼8 h p.i. (murine enteroids) or ∼12 h p.i. (human enteroids), which was due to loss of focus or appearance of infiltrating bacteria that had escaped from neighboring enteroids. The data for each mutant, host species, and dose are based on at least three independent experiments, in which enteroids embedded within the same Matrigel dome were injected with the respective mutant in parallel with *Salmonella* Typhimurium WT. The *P* values in the graphs are based on a log rank test for statistical significance.

We next investigated how TTSS-1 and flagellar motility impact breaching of the epithelial barrier. In our setup, this could be approximated by the basolateral escape of bacteria previously confined within the boundaries of the enteroid (Fig. 1H; also see Fig. S3F and S4G). To this end, human and murine enteroids were microinjected with fluorescently labeled *Salmonella* Typhimurium Δ*invG*, *Salmonella* Typhimurium Δ4 (i.e., Δ*sipA ΔsopBEE2*), or *Salmonella* Typhimurium Δ*motA*, in each case with parallel *Salmonella* Typhimurium WT injections into enteroids within the same dome as controls. Breaching of the epithelial barrier was defined as the point in the time-lapse series where bacterial escape from the enteroid occurred in at least one location (using the Kaplan-Meier model for analysis) (Fig. 4). Bacterial breaching as a rule occurred earlier and was more distinct in murine than in human enteroids (Fig. 4).

TTSS-1-deficient *Salmonella* Typhimurium Δ*invG* breached the epithelial barrier less frequently and at later time points p.i. than the *Salmonella* Typhimurium WT at all doses, with median times of confinement on average ∼1.5 to 4 h longer (Fig. 4A and B). While this difference constituted a trend in human enteroids (Fig. 4A), it was strikingly evident in the more sensitive murine enteroid model, and particularly upon high-dose injection (Fig. 4B). *Salmonella* Typhimurium Δ4 again behaved broadly similarly to *Salmonella* Typhimurium Δ*invG* (significantly longer time of confinement) (Fig. 4C), suggesting that both the TTSS-1 apparatus itself and the TTSS-1 effectors that drive IEC entry promote *Salmonella* Typhimurium breaching of the epithelial barrier in enteroids. Intriguingly, *Salmonella* Typhimurium Δ*motA* showed an even more pronounced delay in the time of confinement (∼2 to 6 h longer than for *Salmonella* Typhimurium WT in parallel infections) and a markedly reduced frequency of bacterial escape events (Fig. 4D and E). These findings held true in both human and murine enteroids and at all three doses tested (Fig. 4D and E). Hence, our combined results suggest that while only TTSS-1 activity boosts luminal colonization, both TTSS-1 activity and flagellar motility promote breaching of the epithelial barrier in *Salmonella* Typhimurium-microinjected enteroids.

### Cycles of IEC invasion, intraepithelial replication, and luminal reemergence fuel *Salmonella* Typhimurium colonization of the enteroid lumen.

**FIG 5:**
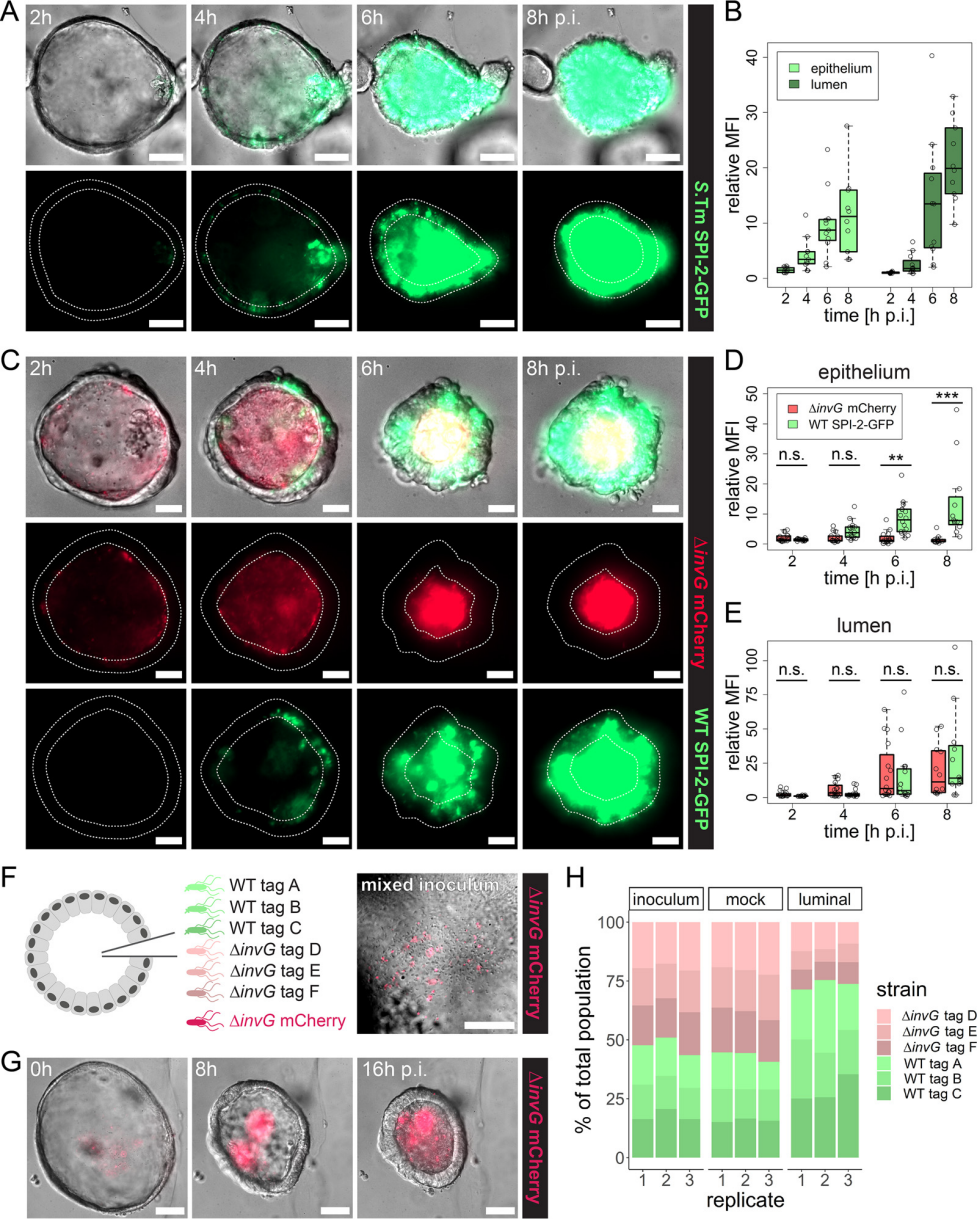
The IEC-invading *Salmonella* Typhimurium population fuels luminal colonization through reseeding. (A and B) Human enteroids were injected with *Salmonella* Typhimurium (*S*.Tm) WT p*ssaG*-GFP at a high dose and imaged as for Fig. 1. Colonization of the epithelial and luminal regions (A) was quantified individually at 2, 4, 6, and 8 h p.i. based on the increase in bacterial fluorescence within each region relative to the initial intensity outside the enteroid. In order to obtain a uniform background signal, a Gaussian blur background subtraction was applied to the image before quantification. The data are based on three independent experiments. (C to E) Human enteroids were injected with a 1:1 mixture of *Salmonella* Typhimurium Δ*invG rpsM*-mCherry and *Salmonella* Typhimurium WT p*ssaG*-GFP at a high dose and imaged as for Fig. 1. Colonization of the epithelial and luminal regions was quantified as for panel B. The data are based on three independent experiments. In the box plots in panels B, D, and E, the height of the boxes represents the IQR, whereas the horizontal line depicts the median. Whiskers extend to the most extreme data point but no further than 1.5× the IQR from the lower (first quartile) or upper (third quartile) boundary of the box. All data points are indicated as circles. MFI, mean fluorescence intensity. Statistical significance in panels D and E was determined by two-way ANOVA with Tukey’s HSD *post hoc* test. n.s., nonsignificant; **, *P* < 0.01; ***, *P* < 0.001. (F to H) Human enteroids were injected with a genetically tagged mixed inoculum consisting of three *Salmonella* Typhimurium WT strains (tags A to C), three *Salmonella* Typhimurium Δ*invG* strains (tags D to F), and *Salmonella* Typhimurium Δ*invG rpsM*-mCherry mixed at a 1:1:1:1:1:1:1 ratio. Injected enteroids were incubated overnight with imaging at 30-min intervals as described for Fig. 1, and luminal *Salmonella* Typhimurium organisms were extracted at ∼16 h p.i. For the mock-injected sample, a fraction of the inoculum (∼500,000 *Salmonella* Typhimurium organisms) was diluted in antibiotic-free human IntestiCult and grown overnight in parallel with the microinjected enteroids. The population structure in the inoculum, mock-injected sample, and luminal population was analyzed by qPCR based on the distribution of the different genetic tags. The percentages shown in panel H are based on the relative abundances that were normalized to *Salmonella* Typhimurium WT tag A in each sample. The data are based on three independent experiments in each of which ∼40 enteroids were injected with 200 to 1,000 *Salmonella* Typhimurium organisms per enteroid (high dose).

To address the causal relationship between TTSS-1-dependent IEC invasion and luminal colonization, we took advantage of the temporal resolution of our microinjection model to determine whether and how the invasive bacterial population contributes to luminal growth. To that end, enteroids were injected with *Salmonella* Typhimurium WT SPI-2–GFP, and the fate of GFP-positive *Salmonella* Typhimurium was followed over time. In accordance with our earlier results (Fig. 1E), GFP-positive foci could be detected within IECs at ∼3 to 4 h p.i. (Fig. 5A). Following intraepithelial expansion, SPI-2–GFP-positive *Salmonella* Typhimurium began reemerging as distinct packages in the enteroid lumen through IEC expulsion (Fig. 5A). Eventually, intraepithelial bacteria released from expelled IECs populated the entire enteroid lumen, filling it completely by ∼6 to 8 h p.i. (Fig. 5A). Quantification of epithelial and luminal fluorescence intensities individually revealed significant bacterial expansion in both compartments (Fig. 5B). Strikingly, the luminal fluorescence intensity was found to exceed the epithelial one at 8 h p.i. (*P* = 0.015) (Fig. 5B). This indicates a contribution of the intraepithelial *Salmonella* Typhimurium population to luminal colonization in enteroids.

To further pinpoint the extent to which reemergence of IEC-residing *Salmonella* Typhimurium can contribute to luminal colonization, enteroid coinfections were performed with a 1:1 mixture of constitutively fluorescent *Salmonella* Typhimurium Δ*invG* (carrying *rpsM*-mCherry) and *Salmonella* Typhimurium WT carrying the SPI-2–GFP construct. As in the single-strain infections, SPI-2–GFP-positive *Salmonella* Typhimurium WT organisms were primarily found within the epithelium at early time points (2 to 4 h p.i.), whereas the *Salmonella* Typhimurium Δ*invG* strain was confined to the enteroid lumen at all time points of the imaging series (Fig. 5C to E). *Salmonella* Typhimurium Δ*invG* fluorescence gradually increased in the lumen (Fig. 5E). Importantly, *Salmonella* Typhimurium WT SPI-2–GFP fluorescence in the lumen accumulated with similar kinetics and potency (Fig. 5E).

At least two mechanisms might account for the contribution of *Salmonella* Typhimurium IEC invasion to enteroid lumen colonization. The cycles of IEC invasion, intraepithelial expansion, and reemergence through IEC expulsion (Fig. 5A to E) could by themselves expand the total luminal pool of *Salmonella* Typhimurium. Alternatively, some consequence of TTSS-1-dependent IEC invasion (e.g., luminal accumulation of IEC debris or hampered antimicrobial peptide [AMP] secretion) could change the conditions for bacterial growth in the lumen itself, which would benefit any *Salmonella* Typhimurium organisms residing in that compartment. To distinguish between these two scenarios, we injected enteroids with a genetically tagged mixed consortium (65), consisting of three invasive *Salmonella* Typhimurium WT strains (tags A to C) and three noninvasive *Salmonella* Typhimurium Δ*invG* strains (tags D to F), along with a fluorescently (*rpsM*-mCherry) labeled *Salmonella* Typhimurium Δ*invG* tracer strain for visualization (Fig. 5F). Luminal *Salmonella* Typhimurium organisms were extracted at ∼16 h p.i., and the population structure was analyzed by real-time quantitative PCR (qPCR), using primers specific for tags A to F.

The enteroid colonization dynamics of the fluorescent *Salmonella* Typhimurium Δ*invG* tracer strain were broadly similar to what was previously observed for the corresponding single-strain infections (Fig. 5G; Fig. S10A), and plating to determine the CFU further confirmed successful expansion of the genetically tagged luminal *Salmonella* Typhimurium population (Fig. S10B). Bacterial escape was observed in ∼45% of the enteroids and, as anticipated, mainly involved *Salmonella* Typhimurium WT tag A to C strains, but expansion of the escaper population was efficiently suppressed by gentamicin added to the surrounding medium (Fig. S10A to C). Importantly, the population structure of the luminal *Salmonella* Typhimurium population revealed a consistent increase in the abundance of all *Salmonella* Typhimurium WT strains by 16 h p.i., whereas the relative strain abundances in mock infection samples (overnight growth of the mixed consortium in IntestiCult medium in the absence of enteroids) remained unchanged (Fig. 5H). These results replicate the ∼2- to 3-fold colonization advantage observed for the *Salmonella* Typhimurium WT strain in single-strain injections (compare Fig. 5H with Fig. 2D, F, and H). The fact that the growth advantage of *Salmonella* Typhimurium WT was maintained in coinfections with lumen-confined *Salmonella* Typhimurium Δ*invG* refutes the idea that an altered luminal environment can explain the link between TTSS-1 and luminal colonization. Altogether, our findings demonstrate instead that a cycle(s) of TTSS-1-dependent IEC invasion, intraepithelial replication, and reemergence through IEC expulsion potently complements planktonic *Salmonella* Typhimurium growth for colonization of the enteroid lumen.

10.1128/mBio.02684-20.10 FIG S10 Barcoded infection confirms that *Salmonella* Typhimurium TTSS-1 promotes breaching of the epithelial barrier in enteroids. Barcoded microinjection and qPCR analysis were performed as for Fig. 5F to H, and the escaped bacterial population was recovered from the medium surrounding the dome that contained the microinjected enteroids. (A) *Salmonella* Typhimurium (*S*.Tm) luminal expansion and breaching of the epithelial barrier during overnight incubation (∼16 h) of enteroids injected with the mixed inoculum. Arrowheads indicate the position of bacterial escape. Please note that growth of the escaped population was efficiently inhibited by gentamicin (6 μg/ml) added to the surrounding medium. (B) CFU recovered from the enteroid lumen (luminal) and the medium surrounding the dome that contained the microinjected enteroids (escaped). The grey area indicates the estimated number of bacteria injected per experiment (40 enteroids × 200 to 1,000 *Salmonella* Typhimurium organisms/enteroid = 8,000 to 40,000 *Salmonella* Typhimurium organisms). (C) Population structure of the escaped population in comparison with the inoculum. The inoculum data are replotted from Fig. 5H, and the data are based on two independent experiments in each of which ∼40 enteroids were injected with 200 to 1,000 *Salmonella* Typhimurium per enteroid (high dose). Download FIG S10, PDF file, 1.1 MB. Copyright © 2021 Geiser et al. 2021 Geiser et al. This content is distributed under the terms of the Creative Commons Attribution 4.0 International license.

## DISCUSSION

Recent studies have employed microinjection of human PS-derived intestinal epithelial organoids (42, 43) and murine enteroids (46, 47) to study individual aspects of *Salmonella* Typhimurium infection. While the PS-derived organoid microinjection model has confirmed *Salmonella* Typhimurium IEC invasion and establishment of an intracellular niche as two central infectious events (42), our study provides a description of the entire early *Salmonella* Typhimurium infection cycle, with all its successive steps, in both human and murine enteroids (Fig. 1; Fig. S3 and S4). Along with an earlier description of the *Cryptosporidium* infection cycle in human enteroids (58), this establishes microinjection of mammalian 3D enteroids as a versatile tool for time-resolved, multicompartment studies of both prokaryotic and eukaryotic gut infections. Moreover, the high temporal resolution of the enteroid microinjection model offers an important advantage over *in vivo* infection models, which has also been exploited by others to, e.g., trace AMP secretion by murine Paneth cells in response to *Salmonella* Typhimurium (47).

*Salmonella* Typhimurium employs flagellar motility to navigate the gut lumen and reach the epithelium (3, 4, 6). Our single-particle tracking shows that *Salmonella* Typhimurium can move relatively unconstrained in the lumen of enteroids, reach the IEC surface within seconds, and engage in near-surface swimming (Fig. 1A to C). In addition, motility promotes breaching of the epithelial barrier at IEC invasion foci in human as well as murine enteroids later in the infection (Fig. 4D and E). Notably, however, gravitational sedimentation also permits flagellum-independent IEC invasion to occur specifically at the enteroid bottom plane (Fig. 3F).

Our results further reveal an impact of *Salmonella* Typhimurium TTSS-1 on IEC invasion, breaching of the epithelial barrier, and lumen colonization (Fig. 2 and 4A to C; Fig. S6, S7, and S8). The first two effects were anticipated and confirm numerous previous reports of observations made across model systems (9, 11, 15, 17, 23, 25, 31, 67). However, the attenuated luminal colonization by noninvasive strains (*Salmonella* Typhimurium Δ*invG* and *Salmonella* Typhimurium Δ4) (Fig. 2; Fig. S6 and S8) was unexpected, as these strains exhibit no growth defect in broth culture (65). Nevertheless, this poor colonization of the lumen agrees with an earlier study of noninvasive *Salmonella* Typhimurium infection in microinjected murine enteroids (46). That and other studies have verified the presence of AMPs in the murine enteroid and human PS-derived organoid lumen and established a contribution of AMPs to restricting luminal *Salmonella* Typhimurium expansion (43, 46, 47). In addition, a steep oxygen gradient across the intestinal epithelium, resulting in reduced oxygen levels (68–70) and (partially) anaerobic metabolism of the bacteria in the lumen, might also contribute to the observed submaximal growth rates. Based on *Salmonella* Typhimurium Δ*invG* fluorescence curves (Fig. S5), we estimate initial *Salmonella* Typhimurium doubling times in the enteroid lumen to be on the order of several hours. Doubling times shorten to ∼2 h later in the infection, which could imply that growth-restricting luminal compounds (e.g., AMPs) eventually become out-titrated, or alternatively that the bacteria adapt metabolically to this environment. It should be noted that several additional mechanisms for luminal population restriction (e.g., commensal microbiota competition, soluble IgA coating, and trapping in mucus) are at play in the more complex intact gut (71). Moreover, our results pertain specifically to human and murine jejunal enteroids, but intestinal-segment-specific differences may well exist, as has been noted for other pathogens (61–63).

Last, our study demonstrates a strong link between *Salmonella* Typhimurium IEC invasion and enhanced luminal colonization (Fig. 5). Luminal reentry of live *Salmonella* Typhimurium released from dying IECs has been suggested by others (17, 20). However, the high temporal resolution of the enteroid microinjection model allowed us to track and quantify how a cycle(s) of TTSS-1-driven invasion, intraepithelial replication, and reemergence through infected IEC expulsion potently complements planktonic *Salmonella* Typhimurium growth in the lumen. It was shown previously that conditions of nutrient limitation and high pathogen densities in planktonic culture elicit SPI-1 gene expression and *Salmonella* Typhimurium invasion of IECs (31, 72, 73). Our present results reveal that IEC invasion reciprocally fuels luminal population expansion. This generates a positive feed-forward loop of epithelial invasion and luminal expansion that results in the rapid and efficient colonization of both compartments. Such a positive feed-forward mechanism might prove even more important in the highly competitive ecosystem of the intact gut.

## MATERIALS AND METHODS

### Ethics statement.

Human jejunal enteroids were generated from tissue resected in the course of bariatric surgery, subsequent to each subject’s giving informed consent. Personal data were pseudonymized before further processing of tissue specimens in the laboratory. The procedures were approved by the local governing body (Etikprövningsmyndigheten, Uppsala, Sweden) under license number 2010-157 with addendum 2010-157-1 (2018-06-13). The maintenance of laboratory mice and experimentation involving murine intestinal tissue were approved by the local governing body (Uppsala Djurförsöksetiska Nämnd, Uppsala, Sweden) under license number C6/16.

### *Salmonella* strains, plasmids, and culture conditions.

All strains used in this study had a Salmonella enterica serovar Typhimurium SL1344 background (SB300; streptomycin resistant) (74). Besides the wild type (*Salmonella* Typhimurium WT), the previously described Δ*invG* (64) and Δ*sipA ΔsopBEE2* (referred to here as *Salmonella* Typhimurium Δ4) mutants (66) were used. The Δ*motA* mutant was generated via transfer of a previously described deletion (75) from a *Salmonella* Typhimurium 14028 strain (C1172) to the SL1344 background by P22 transduction. Chloramphenicol-resistant, isogenically tagged *Salmonella* Typhimurium WT (tags A to C) and *Salmonella* Typhimurium Δ*invG* (tags D to F) strains were used in an earlier study (65). The pFPV-mCherry (*rpsM*-mCherry; Addgene plasmid number 20956) (60), pM975 (p*ssaG*-GFPmut2) (15, 22), and pZ1400 (p*uhpT*-GFP) (18) reporter plasmids were previously used and validated. For infections, *Salmonella* Typhimurium cultures were grown overnight for 12 h in LB–0.3 M NaCl (Sigma-Aldrich) with appropriate antibiotics, followed by subculturing in the same medium without antibiotics at a 1:20 dilution for 4 h. Prior to microinjection, the inoculum was reconstituted in antibiotic-free complete human or mouse IntestiCult medium (StemCell) at a concentration of 5 × 10^8^ to 1 × 10^9^ CFU/ml.

### Human and murine enteroid establishment.

Human jejunal enteroid cultures were established from tissue resected during bariatric surgery performed on otherwise healthy subjects. After resection, the tissue was transported in ice-cold phosphate-buffered saline (PBS; Gibco) until it was opened and fastened to a Styrofoam cushion. Particulate material was removed by washing with cold PBS, and surgical scissors were used to separate the mucosa from the muscle layer. An ∼6- by 6-mm tissue piece excised from the mucosa was washed several times with PBS, minced with surgical scissors, and passed through a 1-ml pipette tip. The minced mucosa was centrifuged and washed once more with cold PBS before incubation in gentle cell dissociation reagent (StemCell) with gentle shaking on ice for 30 min. Following another centrifugation step and resuspension in cold Dulbecco’s modified Eagle medium (DMEM)–F-12 (Gibco) supplemented with 0.25% bovine serum albumin (BSA; Gibco), epithelial crypts were detached by vigorous pipetting. When the resulting suspension had been passed through a 70-μm cell strainer, the crypt concentration was enumerated. The number of crypts required to yield a density of 250 to 750 crypts/dome were centrifuged, resuspended in Matrigel (Corning; product number 356230)–25% DMEM–F-12 and seeded as 50-μl domes in multiwell plates. After solidification at 37°C for 10 min, complete human IntestiCult supplemented with 10 μM Y-27632 (Sigma-Aldrich) and 100 U/ml penicillin-streptomycin (PenStrep; Gibco) was added. Cultures were maintained in a 5% CO_2_ atmosphere at 37°C, and after the first 2 days in culture, Y-27632 was omitted. From then onward, the medium was exchanged every 3 to 4 days. At day 8 to 10 after establishment, the best-looking enteroids were expanded further using the procedure for continuous enteroid subculturing (see below). Murine enteroids of C57BL/6 jejunal origin were established according to a previously published protocol (76), embedded in 50-μl Matrigel domes containing ∼40% complete mouse IntestiCult, and overlaid with IntestiCult supplemented with PenStrep after solidification. Newly established enteroids were frozen at passage 2 in DMEM–F-12–10% fetal bovine serum (FBS; Thermo Fisher Scientific)–10% dimethyl sulfoxide (DMSO; Sigma-Aldrich) and cryopreserved in liquid nitrogen gas phase.

### Human and murine enteroid culture.

For maintenance culturing, both newly generated human and murine enteroids, as well as previously described murine jejunal enteroids (C57BL/6 background) (76), were thawed from cryopreserved stocks and embedded in 50 μl Matrigel domes as described above. After the domes had been allowed to solidify for 10 min at 37°C, they were overlaid with complete human or mouse IntestiCult supplemented with PenStrep. During the first 2 to 3 days after thawing, the culture medium additionally contained 10 μM Y-27632. Cultures were maintained at 37°C in 5% CO_2_, and fresh medium was added every 2 to 3 days. Enteroids were passaged at a 1:3 to 1:12 splitting ratio every 5 to 10 days by breaking up the Matrigel domes through extensive pipetting and incubation in gentle cell dissociation reagent with rocking at 20 rpm. The extracted enteroid fragments were washed once in DMEM–F-12–0.25% BSA and re-embedded in Matrigel domes as described above. Enteroids from passages 4 to 30 were used for experimentation.

### Enteroid microinjection.

*Salmonella* Typhimurium microinjection into human and murine enteroids was performed 4 to 5 days (human enteroids) or 2 to 3 days (murine enteroids) after enteroids had been passaged and embedded in 50-μl elongated, loaf-shaped, ∼90 to 100% Matrigel domes seeded in a 35-mm glass-bottom dish (no. 1.5 coverslip; 20-mm glass diameter, uncoated; MatTek P35G-1.5-20-C). The culture medium was replaced with antibiotic-free complete human or mouse IntestiCult prior to infection. For microinjections of barcoded *Salmonella* Typhimurium consortia, the medium was replaced with complete human IntestiCult containing 6 μg/ml gentamicin. Microinjection needles were generated from 1.0-mm filamented glass capillaries (World Precision Instruments; no. BF100-78-10; Borosilicate, 1 mm wide, 100 mm long, with filament) using a micropipette puller (Sutter Instruments; P-1000; settings: heat = ramp + 5; pull = 60; velocity = 80; delay = 110; pressure = 200) beveled at a 30° angle on a fine-grit diamond lapping wheel. Needles were loaded with the prepared inoculum by fluidic force and mounted on a microinjector (MINJ-FLY; Tritech Research) in a micromanipulator (uMP-4; Senapex). A 0.02- to 0.2-s air pressure pulse was applied to inject enteroids with the respective dose of *Salmonella* Typhimurium. The infectious dose was in each case estimated by eye, based on the number of fluorescent particles emerging from the needle.

### Barcoded-consortium microinjection.

For barcoded-consortium infections, bacterial subcultures of three tagged *Salmonella* Typhimurium WT (tags A to C) and three tagged *Salmonella* Typhimurium Δ*invG* (tags D to F) strains, as well as one fluorescently labeled *Salmonella* Typhimurium Δ*invG* strain (*rpsM*-mCherry), were prepared as described above, mixed at a 1:1:1:1:1:1:1 ratio, and reconstituted in antibiotic-free complete human IntestiCult. Microinjection of ∼40 enteroids per replicate with a total number of ∼200 to 1,000 *Salmonella* Typhimurium organisms per enteroid was performed as described above. Microinjected enteroids were incubated at 37°C and 5% CO_2_ for ∼16 h. For the mock-injected sample, 1 μl of the mixed consortium inoculum was added to a 35-mm glass-bottom dish containing 2 ml antibiotic-free complete human IntestiCult, and the dish was incubated in parallel with the microinjected enteroids. Following overnight incubation, the medium surrounding the Matrigel dome was removed from the dish and saved for enrichment of the escaped population. The dome containing the injected enteroids was washed three times in prewarmed DMEM–F-12 before the enteroids were extracted from the Matrigel by gentle pipetting in ice-cold DMEM–F-12–0.25% BSA using cut pipette tips. After two washes in ice-cold DMEM–F-12–0.25% BSA, the enteroids were broken up mechanically by vigorous pipetting, and luminal bacteria were harvested in 3 ml LB containing 12.5 μg/ml chloramphenicol (Cm; Sigma-Aldrich). For CFU plating, bacterial suspensions extracted from the enteroid lumen and the microinjection supernatant were serially diluted and plated on LB agar containing 12.5 μg/ml Cm.

### Tag quantification by quantitative PCR.

For tag quantification, the bacterial populations recovered from the organoid lumen and microinjection supernatant, as well as a 1:3,000 dilution of the inoculum and mock-injected samples, were enriched for 15 h in 3 ml LB containing 12.5 μg/ml Cm. Half of the enrichment culture was used for genomic DNA extraction using the GenElute bacterial genomic DNA kit (Sigma-Aldrich). Quantitative PCR analysis with the Maxima SYBR green/ROX qPCR master mix (2×) (Thermo Fisher Scientific) was performed on a Bio-Rad CFX 384 instrument using 9 ng of genomic DNA (gDNA) and tag-specific primers as previously described (65, 77). The relative abundances of all strains were calculated as 2^−Δ^*^CT^*, where Δ*C_T_* was defined as the difference in cycle threshold (*C_T_*) value compared to that of *Salmonella* Typhimurium WT tag A in the same sample (i.e., the relative abundance of *Salmonella* Typhimurium WT tag A in each sample was set to 1). To express the population structure as a percentage, the summed relative abundances of all strains in each sample were set to 100%.

### Time-lapse microscopy.

Microinjected enteroids were imaged on a custom-built microscope based on an Eclipse Ti2 body (Nikon), using a 60×, 0.7 numerical aperture Plan Apo Lambda air objective (Nikon) and a back-lit sCMOS (scientific complementary metal oxide semiconductor) camera with a pixel size of 11 μm (Prime 95B; Photometrics). The microscope chamber was maintained at 37°C in a moisturized 5% CO_2_ atmosphere. Bright-field images were acquired using differential interference contrast (DIC), and fluorescence was imaged using the excitation light engine Spectra-X (Lumencor) and emission collection through a quadruple band pass filter (89402; Chroma). For bacterial tracking, the microinjected enteroids as well as a 1:700 to 1:1,000 dilution of the inoculum were imaged at 300- to 500-ms intervals for 20 frames in total. To quantify the initial fluorescence intensity, each enteroid was imaged at the middle plane immediately after microinjection. Live imaging of microinjected enteroids at the middle and/or bottom plane started 10 to 120 min p.i., and time-lapse images were acquired every 5 min for up to 12 h (human enteroids), every 3 min for up to approximately 8 h (murine enteroids), or every 30 min for 16 h (barcoded infections). Confocal (*rpsM*-mCherry) and wide-field (p*ssaG*-GFP) z-stacks of microinjected enteroids were acquired immediately after microinjection as well as at 1.5 to 2 h p.i. (*rpsM*-mCherry) using an X-Light V2 L-FOV spinning-disk module with a pinhole size of 60 μm (CrEST Optics) or at 4 h p.i. (p*ssaG*-GFP), respectively. Time-lapse movies related to the figures can be found at https://doi.org/10.17044/scilifelab.12998570.

### Image analysis.

For motility analysis, single bacteria in the inoculum and within the lumen of microinjected enteroids were tracked using the TrackMate plugin (78) in Fiji (a version of ImageJ) (79). Relative fluorescence intensities were determined in Fiji by manually outlining the enteroid cross-section at the middle plane for each time point and quantifying the fluorescence within this area at 30-min intervals, whereby the fluorescence was normalized to the initial intensity immediately after microinjection for the respective enteroid. Fluorescence intensity profiles were determined in Fiji. Background subtraction in confocal and wide-field z-stack images was also performed in Fiji. The time point of bacterial escape from the enteroid lumen was defined as the time p.i. when the first visible fluorescent *Salmonella* Typhimurium were observed outside the epithelial boundary. For quantification of separate epithelial and luminal fluorescence intensities, the epithelial and luminal regions were defined based on manual measurements of the epithelial thickness and definition of the enteroid outline at the middle plane for each enteroid at 0, 2, 4, 6, and 8 h p.i. Next, a Gaussian blur filter was applied with a standard deviation of 5 μm, and the Gaussian blur was subtracted from the image to reach a uniform background signal close to 0 in the epithelial, luminal, and outside regions. The fluorescence intensity in each region (epithelium, lumen, and outside) was then quantified, and epithelial and luminal fluorescence intensities were normalized to the outside fluorescence intensity at 0 h p.i. for the respective enteroid.

### Statistical analysis.

Where applicable, statistical significance was determined by two-way analysis of variance (ANOVA) with Tukey’s honestly significant difference (HSD) *post hoc* test applying the functions aov() and TukeyHSD() in RStudio (80). For analysis of bacterial escape from the enteroid lumen, survival analysis according to the Kaplan-Meier model was performed using the functions Surv(), survfit(), and survdiff() in the survival package for RStudio (81), and statistical significance was assessed by the log rank test.
