# Supplementary figures and images for: Salmonella enterica Serovar Typhimurium Exploits Cycling through Epithelial Cells To Colonize Human and Murine Enteroids
Source: mBio. 2021 Jan 12;12(1):e02684-20. doi: 10.1128/mBio.02684-20 (PMC7844539; doi:10.1128/mBio.02684-20)

Supplementary Figure 1

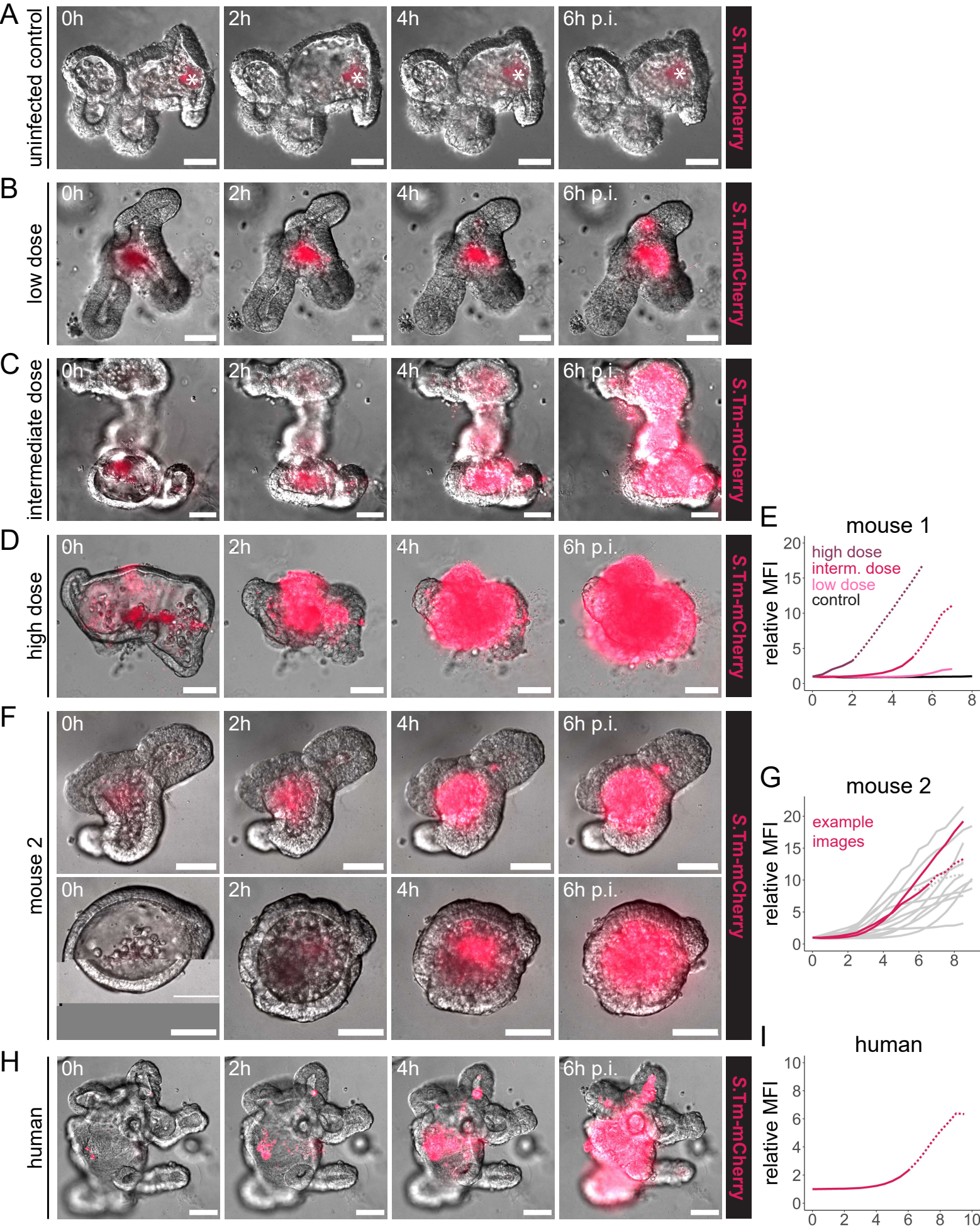

Supplement: FIG S1 [file mBio.02684-20-sf001.pdf]

Supplementary Figure 2

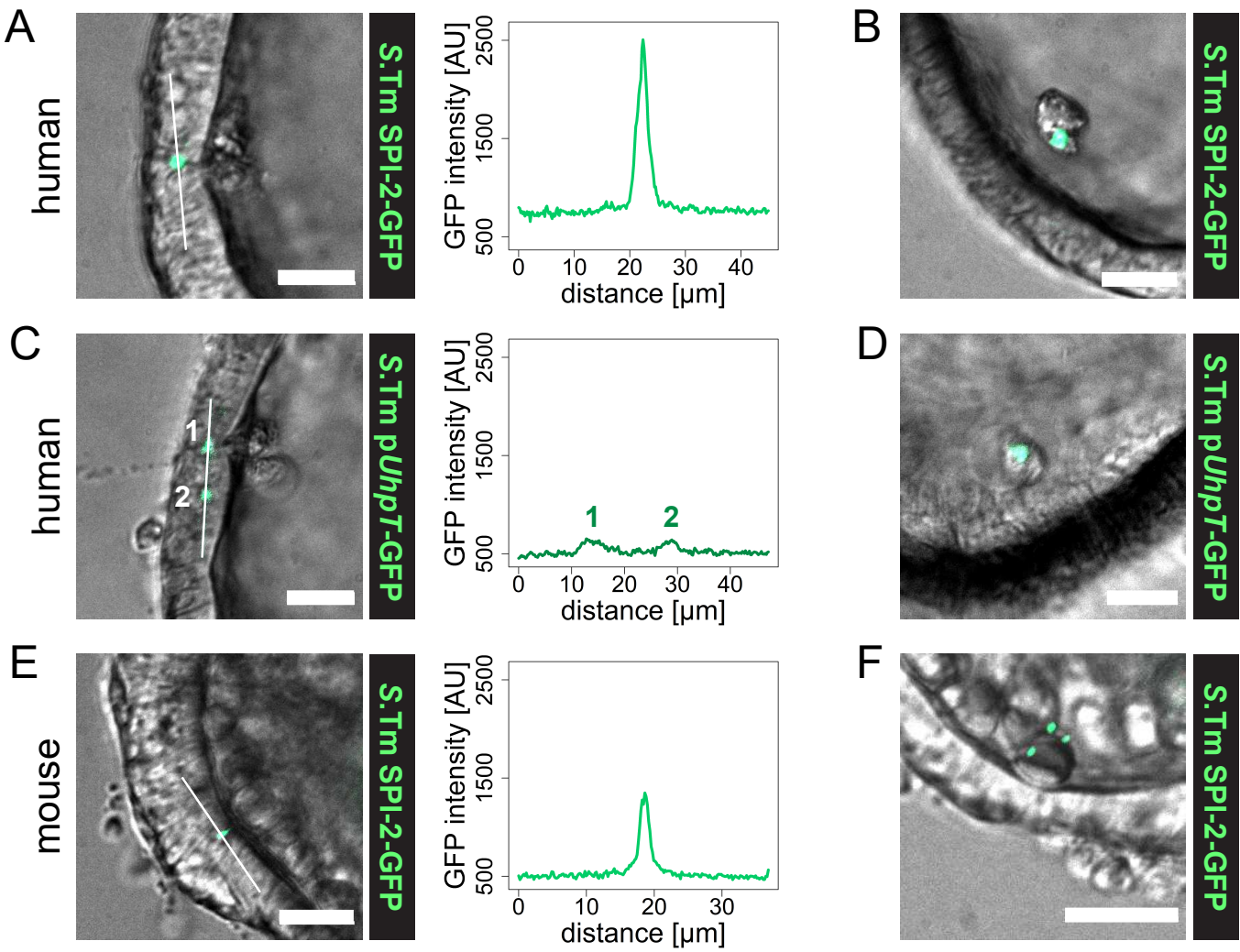

Supplement: FIG S2 [file mBio.02684-20-sf002.pdf]

Supplementary Figure 3

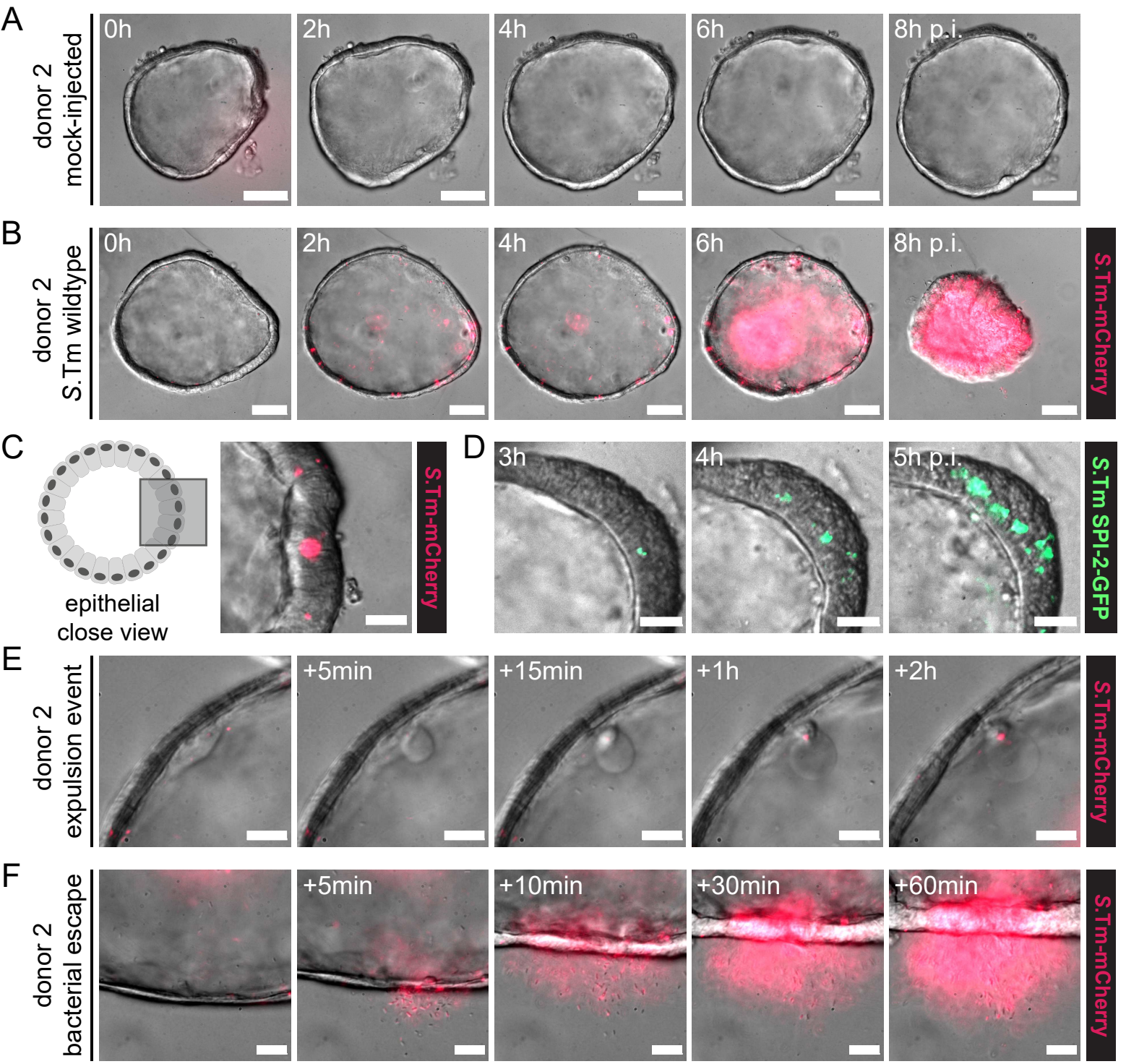

Supplement: FIG S3 [file mBio.02684-20-sf003.pdf]

Supplementary Figure 4

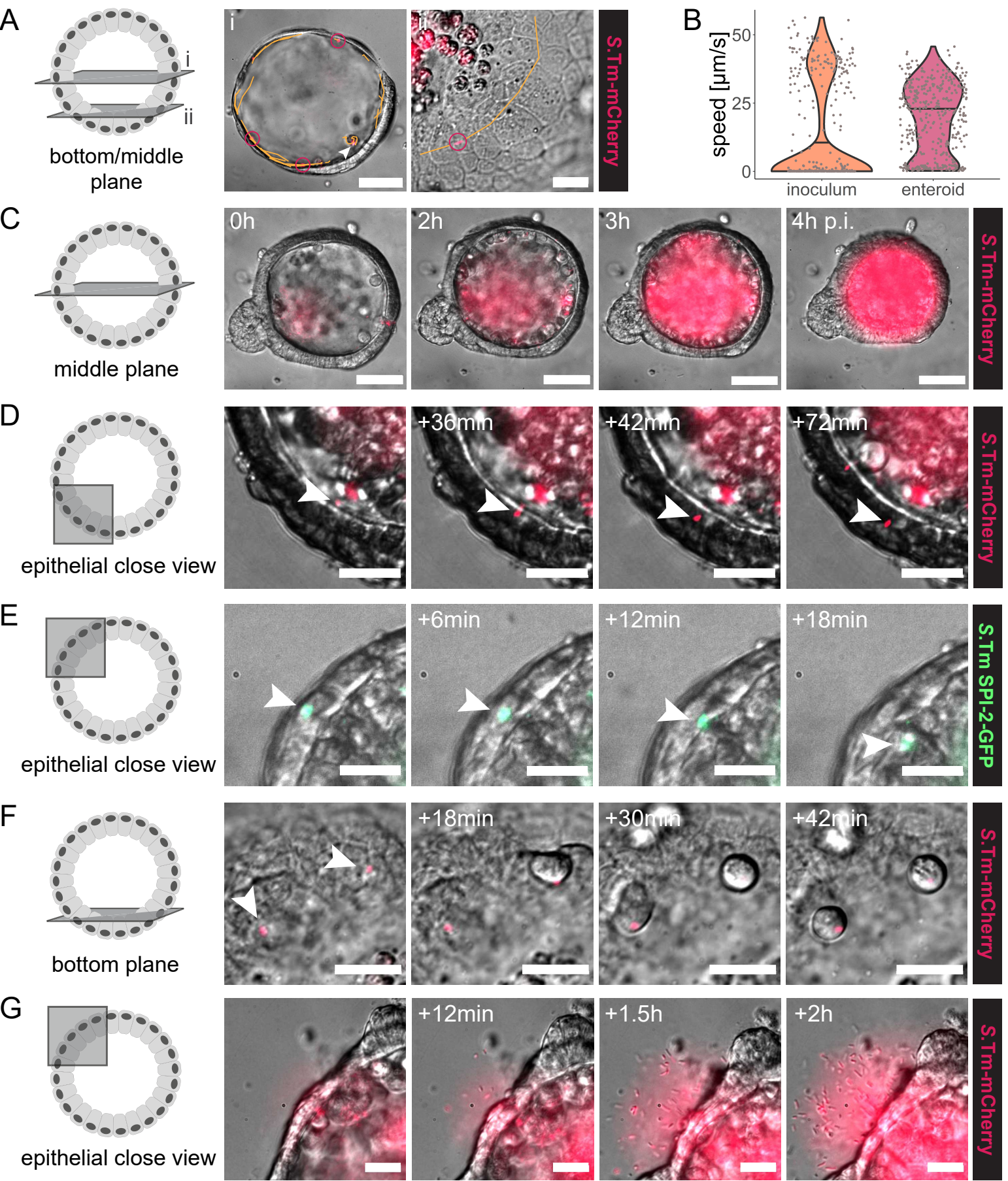

Supplement: FIG S4 [file mBio.02684-20-sf004.pdf]

Supplementary Figure 5

A

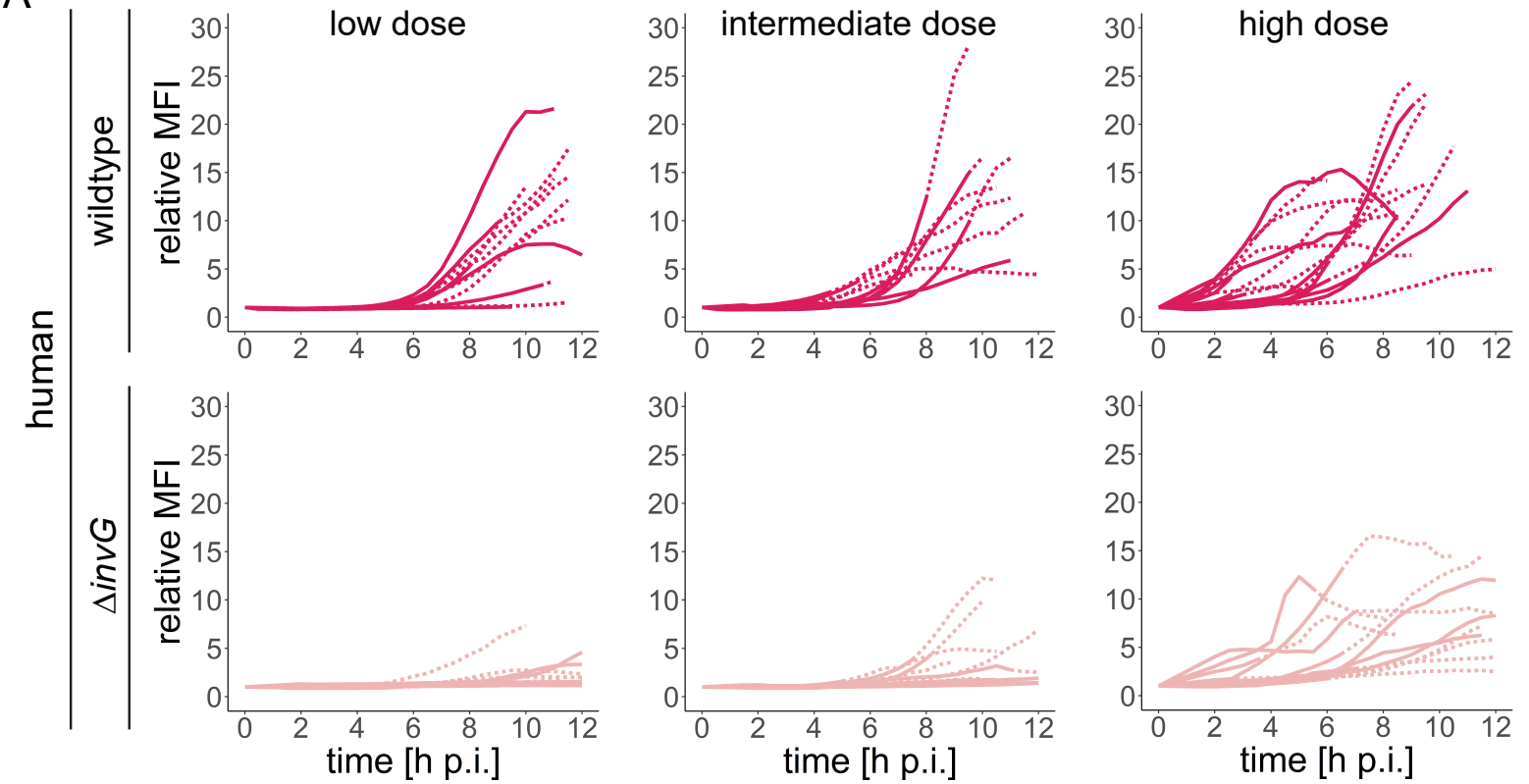

B

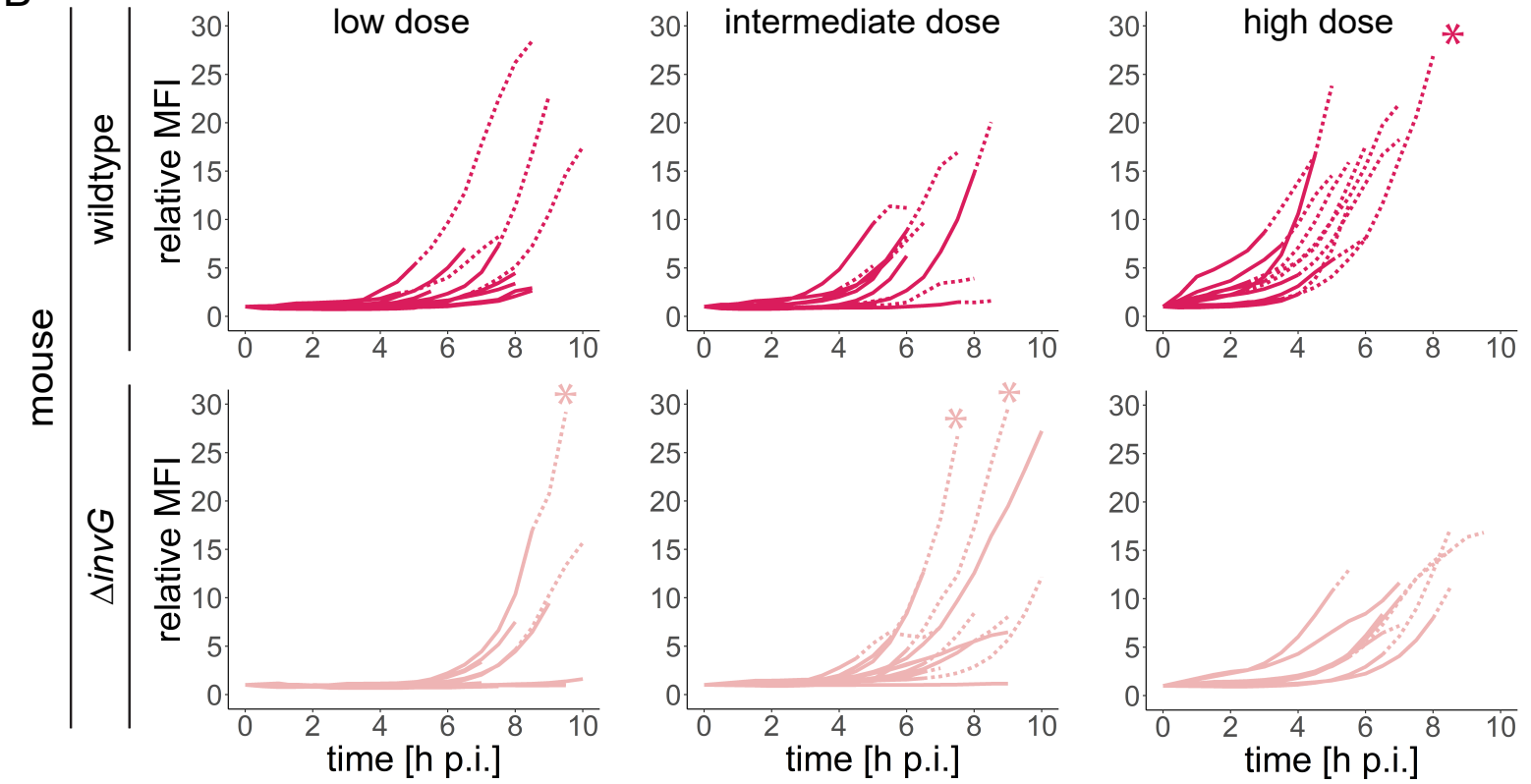

Supplement: FIG S5 [file mBio.02684-20-sf005.pdf]

Supplementary Figure 6

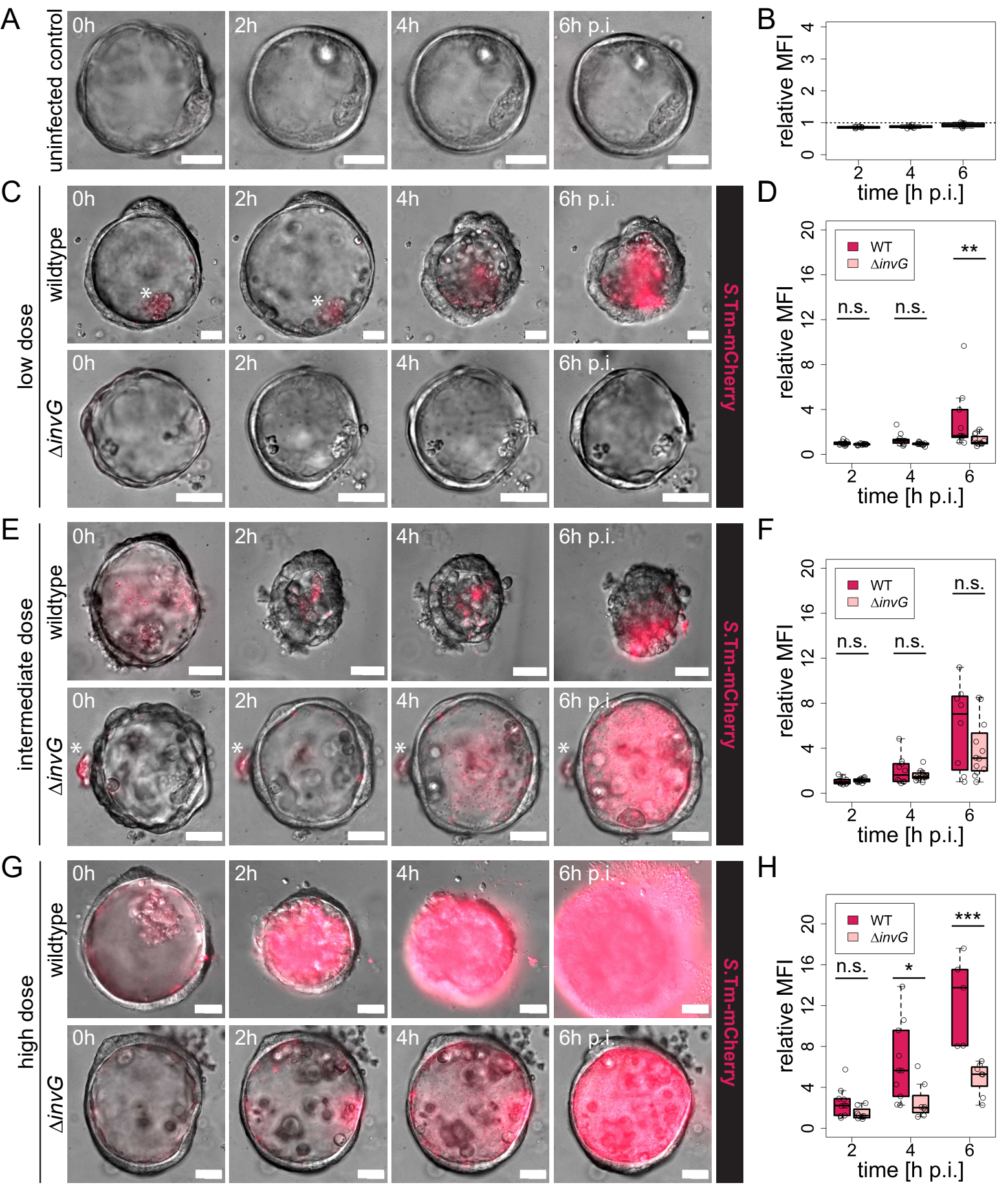

Supplement: FIG S6 [file mBio.02684-20-sf006.pdf]

Supplementary Figure 7

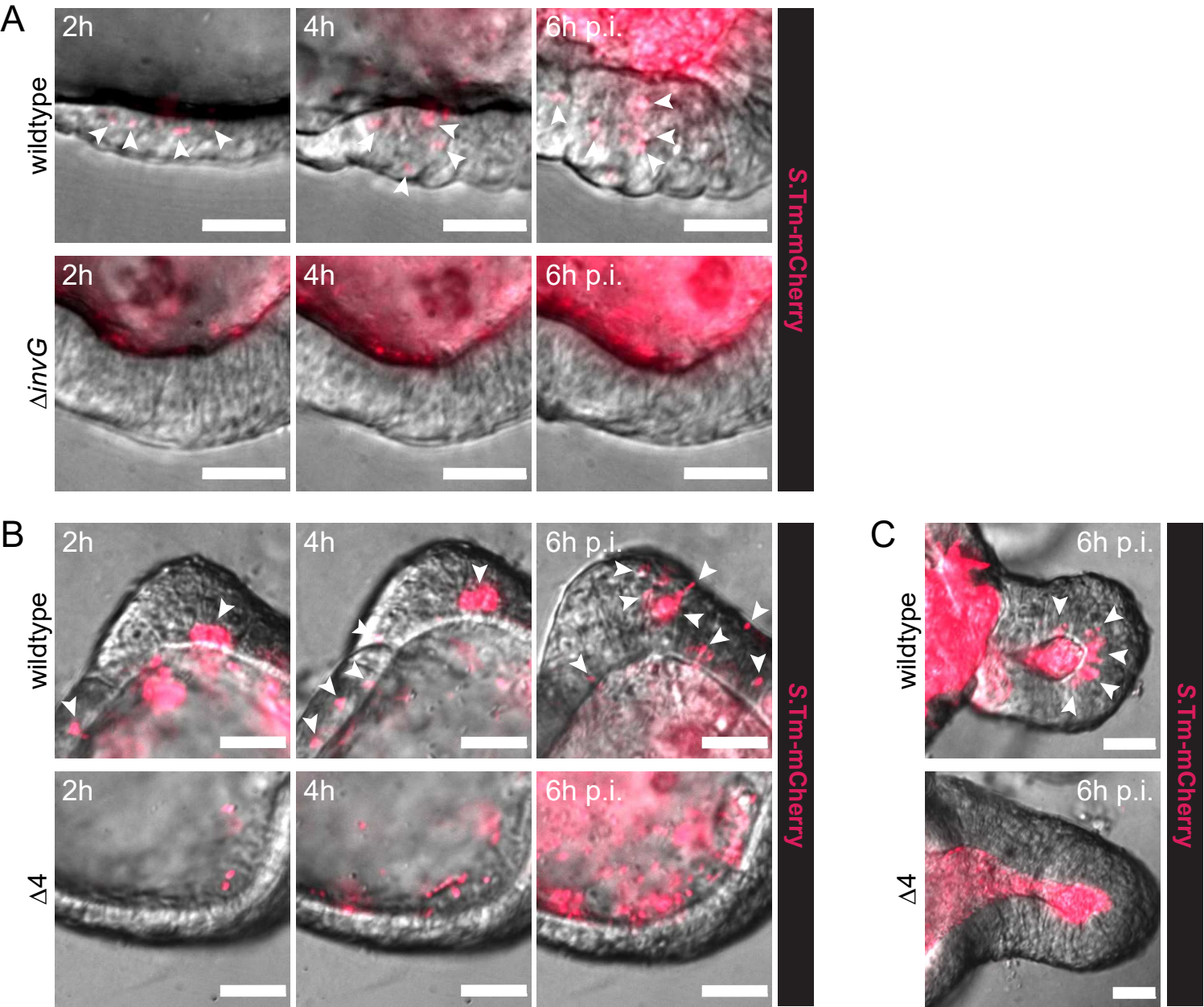

Supplement: FIG S7 [file mBio.02684-20-sf007.pdf]

Supplementary Figure 8

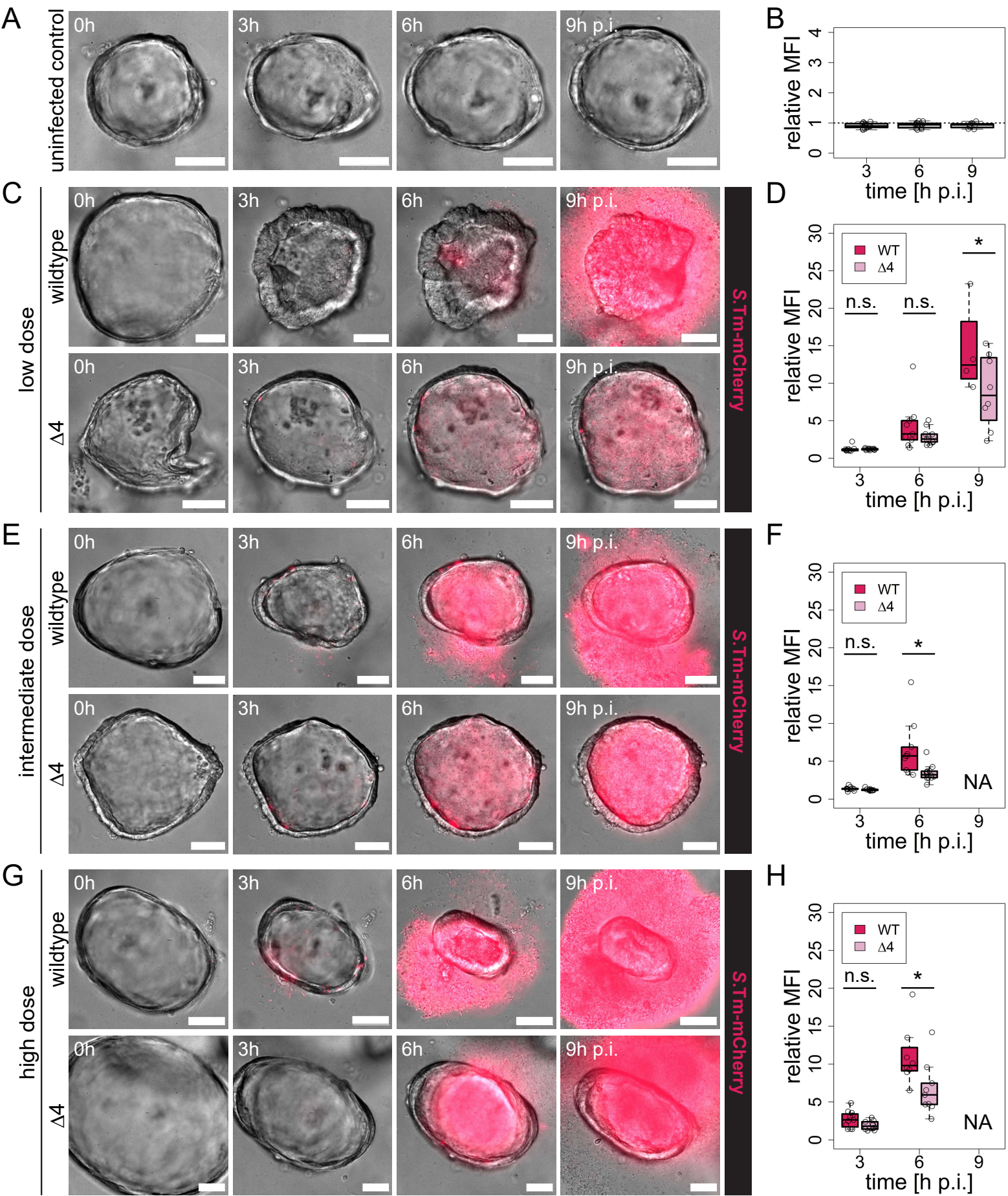

Supplement: FIG S8 [file mBio.02684-20-sf008.pdf]

Supplementary Figure 9

A

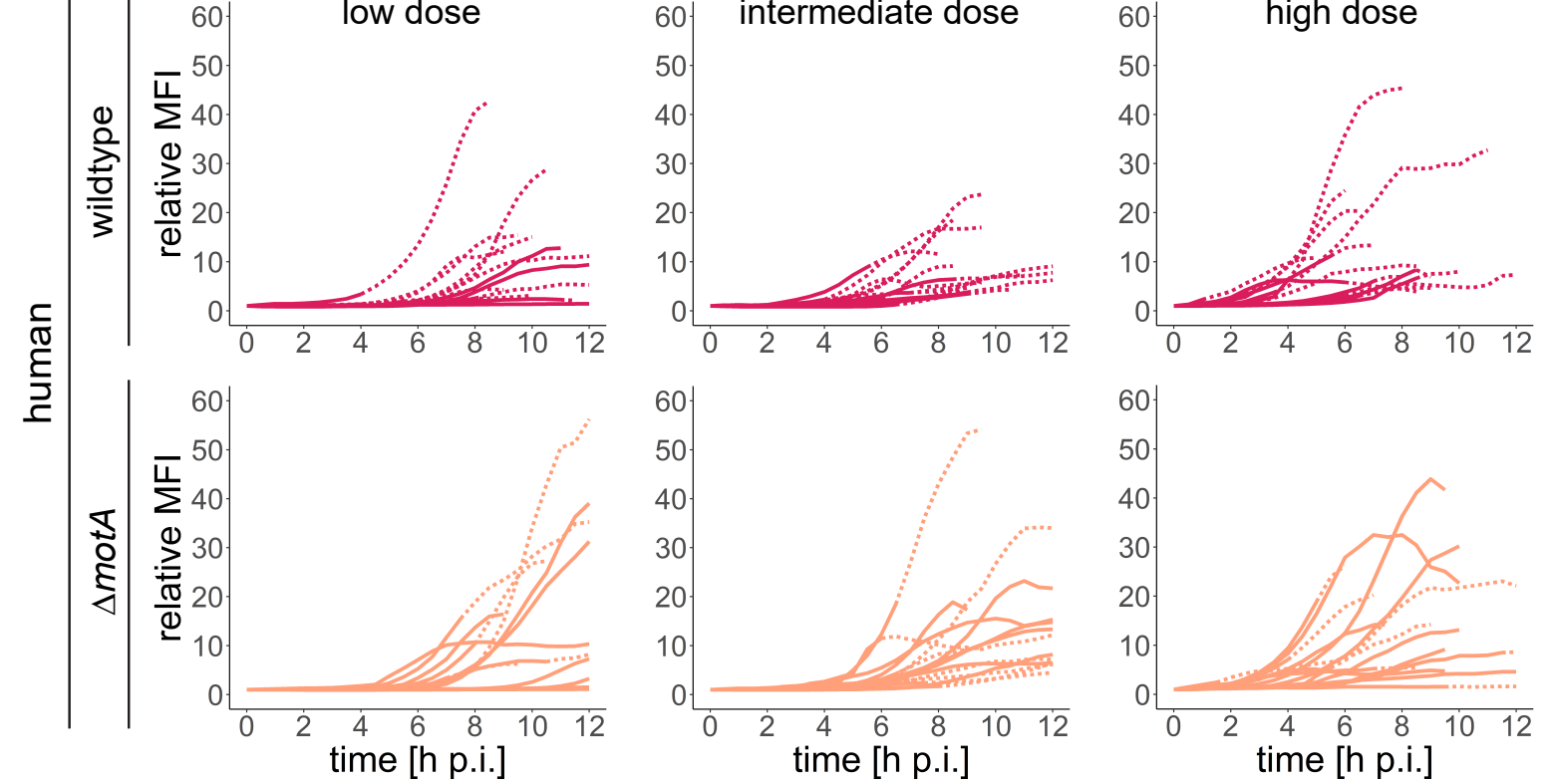

B

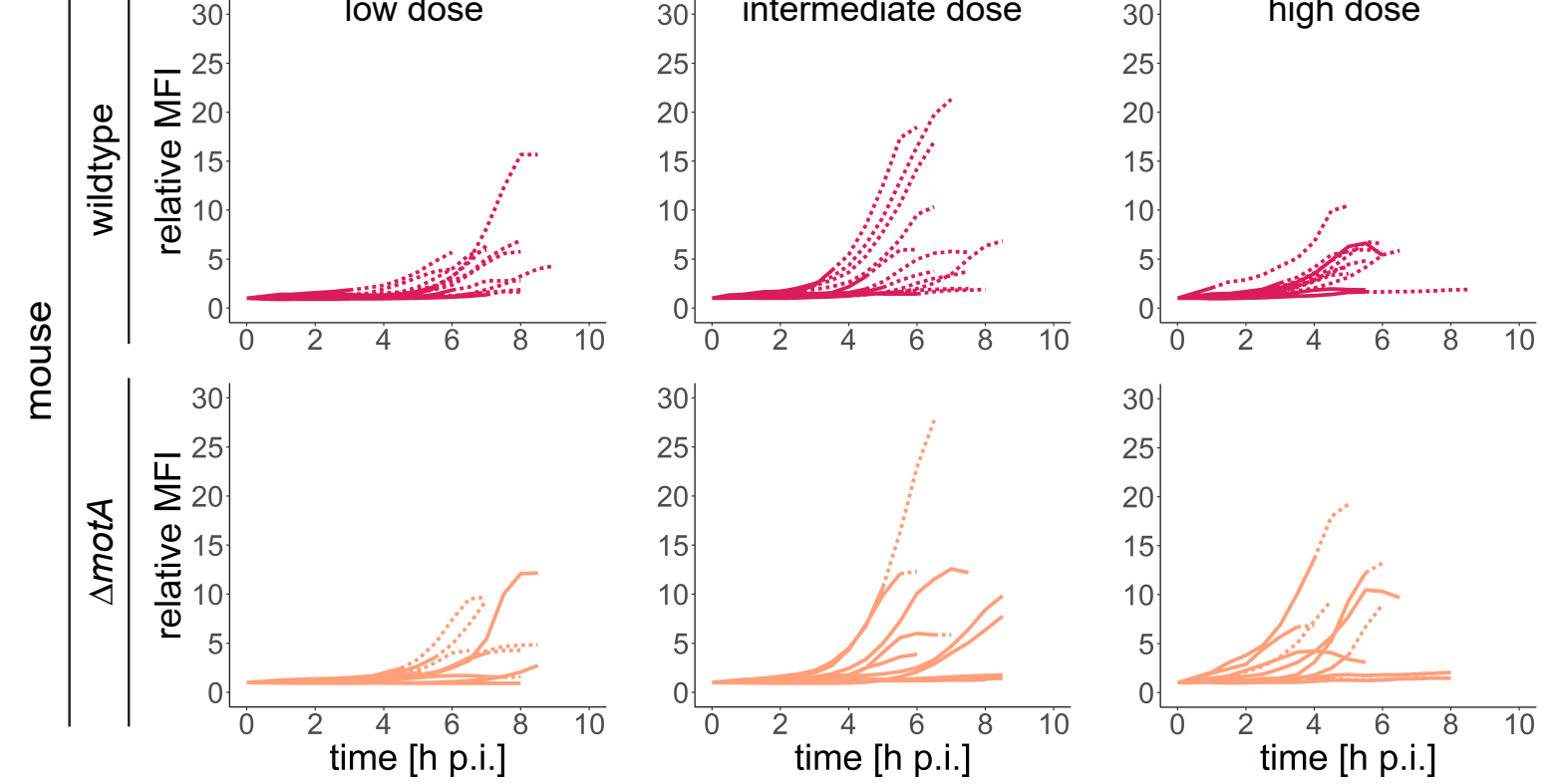

Supplement: FIG S9 [file mBio.02684-20-sf009.pdf]

Supplementary Figure 10

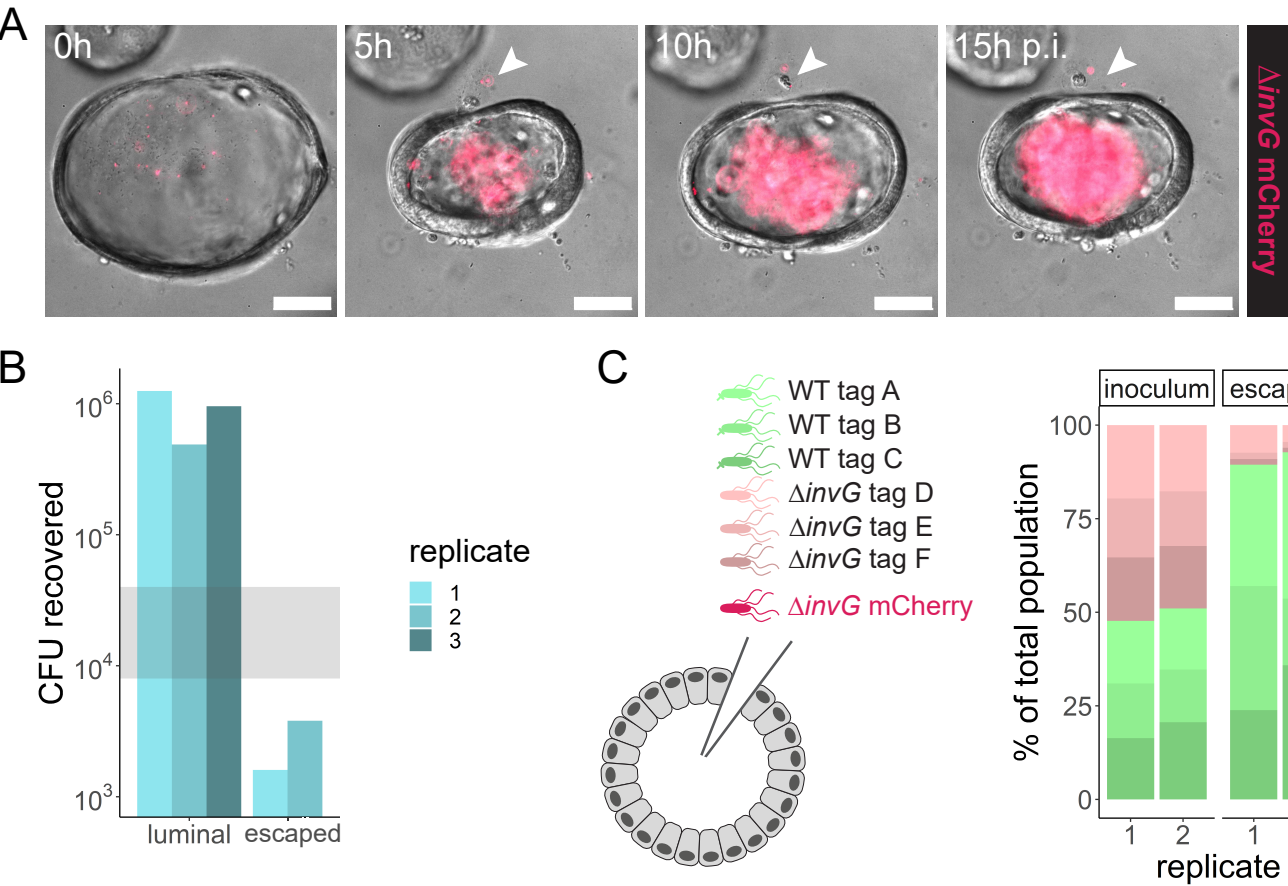

Supplement: FIG S10 [file mBio.02684-20-sf010.pdf]
